# Using Deep Learning to Extrapolate Protein Expression Measurements

**DOI:** 10.1002/pmic.202000009

**Published:** 2020-10-16

**Authors:** Mitra Parissa Barzine, Karlis Freivalds, James C. Wright, Mārtiņš Opmanis, Darta Rituma, Fatemeh Zamanzad Ghavidel, Andrew F. Jarnuczak, Edgars Celms, Kārlis Čerāns, Inge Jonassen, Lelde Lace, Juan Antonio Vizcaíno, Jyoti Sharma Choudhary, Alvis Brazma, Juris Viksna

**Affiliations:** ^1^ European Molecular Biology Laboratory European Bioinformatics Institute EMBL‐EBI Wellcome Trust Genome Campus Hinxton CB10 1SD UK; ^2^ Institute of Mathematics and Computer Science University of Latvia Riga LV1459 Latvia; ^3^ Faculty of Computing University of Latvia Riga LV1586 Latvia; ^4^ Institute of Cancer Research London SW3 6JB UK; ^5^ Computational Biology Unit Informatics Department University of Bergen Bergen NO5020 Norway

**Keywords:** deep learning networks, Gene Ontology, mass spectrometry, protein abundance prediction, UniProt keywords

## Abstract

Mass spectrometry (MS)‐based quantitative proteomics experiments typically assay a subset of up to 60% of the ≈20 000 human protein coding genes. Computational methods for imputing the missing values using RNA expression data usually allow only for imputations of proteins measured in at least some of the samples. In silico methods for comprehensively estimating abundances across all proteins are still missing.

Here, a novel method is proposed using deep learning to extrapolate the observed protein expression values in label‐free MS experiments to all proteins, leveraging gene functional annotations and RNA measurements as key predictive attributes. This method is tested on four datasets, including human cell lines and human and mouse tissues. This method predicts the protein expression values with average $R^{2}$ scores between 0.46 and 0.54, which is significantly better than predictions based on correlations using the RNA expression data alone. Moreover, it is demonstrated that the derived models can be “transferred” across experiments and species. For instance, the model derived from human tissues gave a $R^{2}=0.51$ when applied to mouse tissue data. It is concluded that protein abundances generated in label‐free MS experiments can be computationally predicted using functional annotated attributes and can be used to highlight aberrant protein abundance values.

## 1 Introduction

Mass spectrometry (MS)‐based proteomics is routinely used to measure the abundance of proteins in biological samples. However, due to technical limitations and restrictions in the limits of detection and dynamic range of analyses performed, complete proteomes of complex biological samples are never fully characterized and quantified.^[^ ^1^, ^2^, ^3^ ^]^ Missing protein expression values are a constant confounding factor in the downstream analysis.

**Figure 1:**
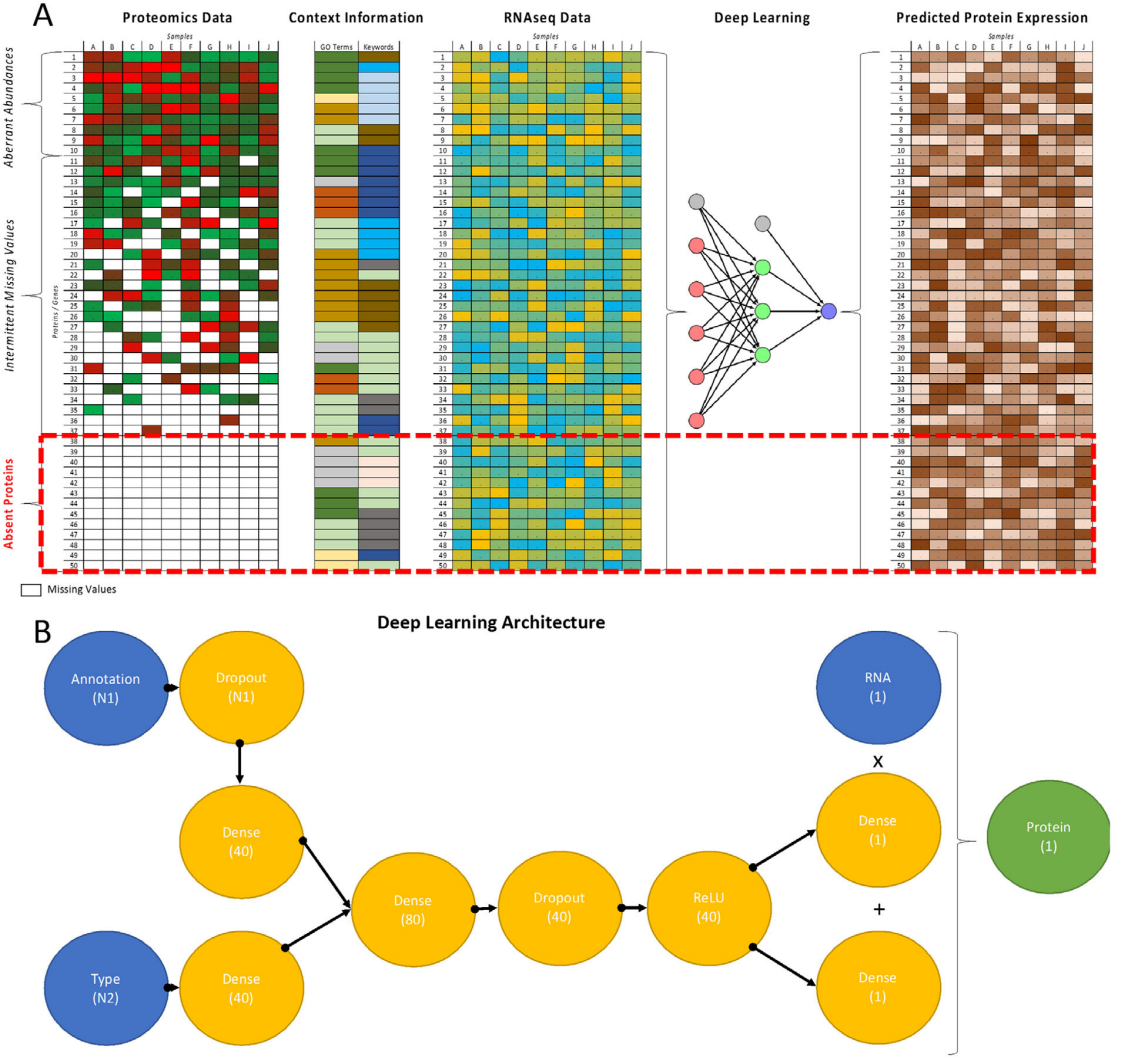
Summary of the approach: a) Using deep learning methods to predict the values for unobserved proteins in MS‐proteomics experiments. Measured protein expression values in combination with context information such as functional annotations and RNAseq measurements are used to train a neural network which can predict protein expression for all proteins, including those not experimentally measured in any sample. b) The deep learning network architecture for predicting the protein abundance values based on RNA and gene annotations: Blue nodes represent inputs, the green nodes outputs, and the orange nodes represent intermediate layers. In parentheses dimensionalities of input and output vectors as well as those of the network layers are shown.

As schematically shown in **Figure** **1**, protein expression missing values can be classified into three categories: i) unobserved proteins, with no identifiable peptides in any samples and/or MS runs; ii) intermittently detected proteins, present in some but not in all experimental samples and/or MS runs; and iii) ambiguous and aberrantly expressed proteins, where it is not possible to distinguish or correctly quantify proteins based on the detected peptides, due to limitations caused by the protein inference. There are various technical and biological explanations for the lack of experimental observation of any given protein. Biological explanations include restricted expression, proteoform complexity, and abundance level. Technical reasons, which are most frequently associated with peptide observability, include peptide ionization, sample complexity, and spectral quality, among others.

To cope with missing protein values in proteomics experiments, various methods have been developed to impute the expression values.^[^ ^4^, ^5^ ^]^ These approaches try to fill intermittent missing values across MS runs using simple or sophisticated models built on a larger set of data. There are four main approaches for imputation of missing values. One is to use multidimensional spectral alignments to match MS1 peaks between runs to increase the number of identifications between runs and assign real intensity values to missing peptides and proteins. The second approach uses fixed constant single‐values, which can be a minimal value, making the assumption that a protein is missing due to low abundance. Alternatively, an average value can be used, with the assumption being that a general 1:1 ratio across runs will cause less bias. The third approach uses local similarity to find peptides or proteins with similar expression profiles within the same dataset and then use these as the basis for estimating missing values. Finally, the missing values can be reconstituted from a global model using methods such as regression, probabilistic principal component analysis, Bayesian component analysis, and data normalization techniques. These sophisticated methods can produce a more nuanced missing value imputation at a higher computational cost.

A major shortcoming of all the current imputation methods is that they only deal with intermittently detected proteins, requiring identification and quantification of the protein in some samples to support the modeling algorithm. With the rise of proteogenomic approaches, it is becoming increasingly common to perform both proteomic MS and RNAseq analysis in the same samples. In fact, several recent large‐scale studies have compared transcript and protein expression. These studies report a wide range in the correlation of protein and transcript expression across different genes, tissues, and samples, which overall is found to be moderately positive, although exact numbers vary enormously.^[^ ^6^, ^7^, ^8^ ^]^ It has been shown that there are certain classes of proteins that are more closely regulated at the transcriptional level than others. Specifically, proteins with highly variable expression such as metabolic and immune‐related proteins often correlate well, whereas slow turn‐over proteins and those in stable complexes often correlate poorly.^[^ ^9^, ^10^, ^11^ ^]^

**Significance Statement**

Proteome of eukaryotic organisms is highly complex and has dynamic range of many orders of magnitude. It is therefore not surprising that despite significant technological advances, proteomic mass spectrometry experiments continue to be limited in coverage and depth compared to genomics. Most proteomics experiments thus quantify only part of the proteome, leaving many proteins “unobserved” even when they may be present in the proteome. Here, we show that using deep learning techniques, we can leverage gene annotation and RNAseq data to extrapolate the available protein abundances to unobserved proteins in a given experiment. Previous studies have applied machine learning methods to impute partial missing values across experiments and to predict protein abundance across similar samples. The approach presented here is the first to attempt to predict unobserved proteins and thus to provide a method for increasing coverage of the proteome, which is an important development for comparative proteogenomic analyses. Additionally our method highlights aberrantly expressed proteins; these can either be due to biological perturbation or technical and annotational issues in the data. Overall, this study further integrates proteomics and genomics data to advance our understanding of their biological relationships.

Recently, prediction of protein expression levels in samples derived from cancer patients has been the subject of an NCI‐CPTAC DREAM proteogenomics challenge.^[^ ^12^, ^13^ ^]^ The teams used linear and non‐linear models to evaluate prediction of proteomes from genomics data and prior information for unobserved samples. However, to the best of our knowledge, none of the existing approaches attempt to estimate the expression of the experimentally unobserved proteins. Here, we close this gap by proposing a novel method that estimates the expression of unobserved (absent) proteins, where no expression values are recorded in any of the given proteomics datasets. Specifically, we describe a novel method leveraging deep learning (DL) to generate a predictive model based on existing measured protein expression values, RNA expression values for all genes in the same or similar samples, and available gene annotations such as Gene Ontology (GO) and UniProt keywords. We apply the DL approach beyond imputation of partially missing values or additional samples to predict all unobserved protein abundances in label‐free MS experiments. The method predicts the protein expression values with average $R^{2}\;$scores between 0.46 and 0.54, which is significantly better than predictions based on correlations using the RNA expression data alone. Moreover, we show that the derived models can be “transferred” across experiments and species. Finally, we demonstrate that such predictive models can highlight aberrant expression patterns and infer abundances for ambiguous proteins more reliably.

## 2 Experimental Section

### 2.1 Datasets and Their Preparation

The label‐free MS experiments used in this study included three human datasets: Tissue13 (13 tissues and 9637 genes, extracted from the reprocessed Draft Human Proteome,^[^ ^14^, ^15^ ^]^ NCI60 (46 cell lines and 8000 genes),^[^ ^16^ ^]^ and Tissue29 (29 tissues and 12 879 genes).^[^ ^17^ ^]^ The experiment also used a label‐free mouse tissue dataset MouseTissue3 (3 tissues and 6591 genes).^[^ ^18^ ^]^ All RNA and proteome datasets were downloaded from EBI's Expression Atlas,^[^ ^19^ ^]^ with proteomics data for Tissue13 (dataset PXD000561 and its re‐analysis PXD002967), NCI60 (PXD005940), and Tissue29 (dataset PXD010154) measurements based on raw data from the PRIDE database.^[^ ^20^, ^21^, ^22^, ^23^ ^]^ For more information on these datasets and references, see Supporting Information.

Both proteomics and RNAseq abundances are represented as log transformations. To deal with zero values in RNAseq data, in the literature, typically a function log(v+c) is used, where v is the value of the measurement on the original scale and c is a constant, common for the entire dataset. As in most other studies, it is used c=1 (the choice of c is not scale invariant, but c=1 is sufficiently small for the ranges of the values included in the datasets considered here).

Two real value matrices were used as input data to our algorithm, where rows correspond to genes/proteins, while columns correspond to samples (tissues or cell lines). The first matrix contained RNA abundance measurement values and it was assumed that every gene and every sample in this matrix had a defined value (i.e., there were no missing or unobserved gene values). The second matrix contained label‐free protein abundances as quantified in the respective datasets, where some elements, including entire rows, may be missing. Unobserved or missing values in the proteomics data were ignored when training the neural network. In addition, genes were annotated by context information, specifically using GO terms or UniProt keywords (KW), where each gene can have more than one term assigned and the same term can be used to annotate several genes. The goal was to extrapolate the protein abundance values to all genes in the matrix using all the available data, as shown in Figure 1.

### 2.2 Computational Experiments

For the prediction of protein abundance values, we used a specially developed DL network. To train the network for a particular subset of genes, the input given was the measured protein abundances, the RNA expression values, and a binary vector for each gene, describing context information (such as GO terms and/or UniProt keywords). The trained network was then used to make protein abundance predictions, which are based on the equations p(g)=α(g)+β(g)×r(g), where p(g) and r(g) are the protein and RNA abundances for each gene g, and the coefficients α(g) and β(g)are computed from the information provided to the network. It should be noted that the coefficients α(g) and β(g)are specific to each gene and are derived from the supplied context information in a non‐linear way. Further details of network architecture are provided in the Supporting Information.

The gene set for each experiment was partitioned randomly into ten subsets. Over ten iterations, ten models were then built using a training set comprising 90% (9 subsets) of the data and a test set using the remaining 10% (single subset). In each iteration, we trained a DL network on all available information (RNA values, sample identification, and functional annotations) for each training set. The protein values in the test set were then predicted by applying the obtained model to the annotations and RNA values of the genes excluded in the training set. By repeating the process excluding a different subset, each time we obtained a predicted abundance value for every protein in the dataset. We call each of these described experiments a *run*. For each dataset, we performed ten runs based on different randomizations of the initial set to validate the stability of the predictions. In total 100 models were generated and applied for each experiment. We also considered the problem of the unobserved protein expression value prediction only using RNA information or excluding it from the model, that is, only using gene annotations.

Depending on the information used for the training, we considered several prediction models: RNA (only), GO, KW, RNA+GO, RNA+KW, RNA+GO+KW, and Randomised (the abbreviations indicate the information that is presented to the DL network). The Randomised model was used as a baseline comparison with the predictions, and was based on RNA and context information being randomly permuted between the genes in the dataset. The predictions were compared to the measured abundance values for proteins in the test set for which the experimental measurements were available.

As a natural benchmark for comparison, we also considered linear regression (LR)‐based predictions from the RNAseq values. Not surprisingly, DL models trained using only RNA and LR model predictions were almost identical (with small fluctuations for RNA between different runs)—without any context information provided to DL network, one can hardly expect better predictions than LR, and the fact that these values largely matched at least partially re‐confirms that the proposed network design is conceptually sound. Thus, we further discuss only LR results because they are a simpler and a more familiar benchmark.

The fact that predictions were based on gene‐specific coefficients α(g) and β(g)allows precomputation of these on one dataset and then testing their predictive value for the same or for homologous genes in different datasets (of the same or different species).

In the case of the dataset *MouseTissue3*, the experiments did not include the models with KW data. However, we tested the inter‐species applicability of the computed gene‐specific coefficients α(g) and β(g)for homologous genes. These coefficients were computed from *Tissue29* for the two tissues it shares with the dataset *MouseTissue3* (liver and testis) using the *RNA+GO+KW* model, and then applied for the prediction of protein abundances for 5388 homologous genes from the *MouseTissue3* dataset. For detailed description see Supporting Information.

### 2.3 Measures of Prediction Accuracy

We assessed the prediction accuracy using the $R^{2}$ score, which has been adapted almost exclusively in all the related work (and in most cases is simply derived from Pearson's correlation coefficient r by taking $R^{2}=r^{2}$). For a given dataset, $R_{\mathrm{Davg}}^{2}$ is the average score over the whole dataset, $R^{2}\left[t\right]$ is the score for a specific tissue (or cell line) t. $R_{\mathrm{Tavg}}^{2}$ denotes the average score over all tissues, and $R_{\mathrm{Max}}^{2}$ and $R_{\mathrm{Min}}^{2}$ refer to the highest and lowest prediction accuracies for tissues within a given dataset. The exact formulas for computation of the $R^{2}$ scores are provided in Supporting Information. We introduce the notions of $R^{2}\left(g,t\right)$, which show the contribution of gene g to the overall prediction accuracy $R_{\mathrm{Davg}}^{2}$, $R^{2}\left[t\right]\left(g\right)$, showing gene contribution to prediction accuracy for specific tissue $R^{2}\left[t\right]$, and $R_{\mathrm{avg}}^{2}\left(g\right)$—average contribution of gene to prediction accuracy over all tissues.

## 3 Results

### 3.1 Predictions on the Human Datasets

**Figure 2:**
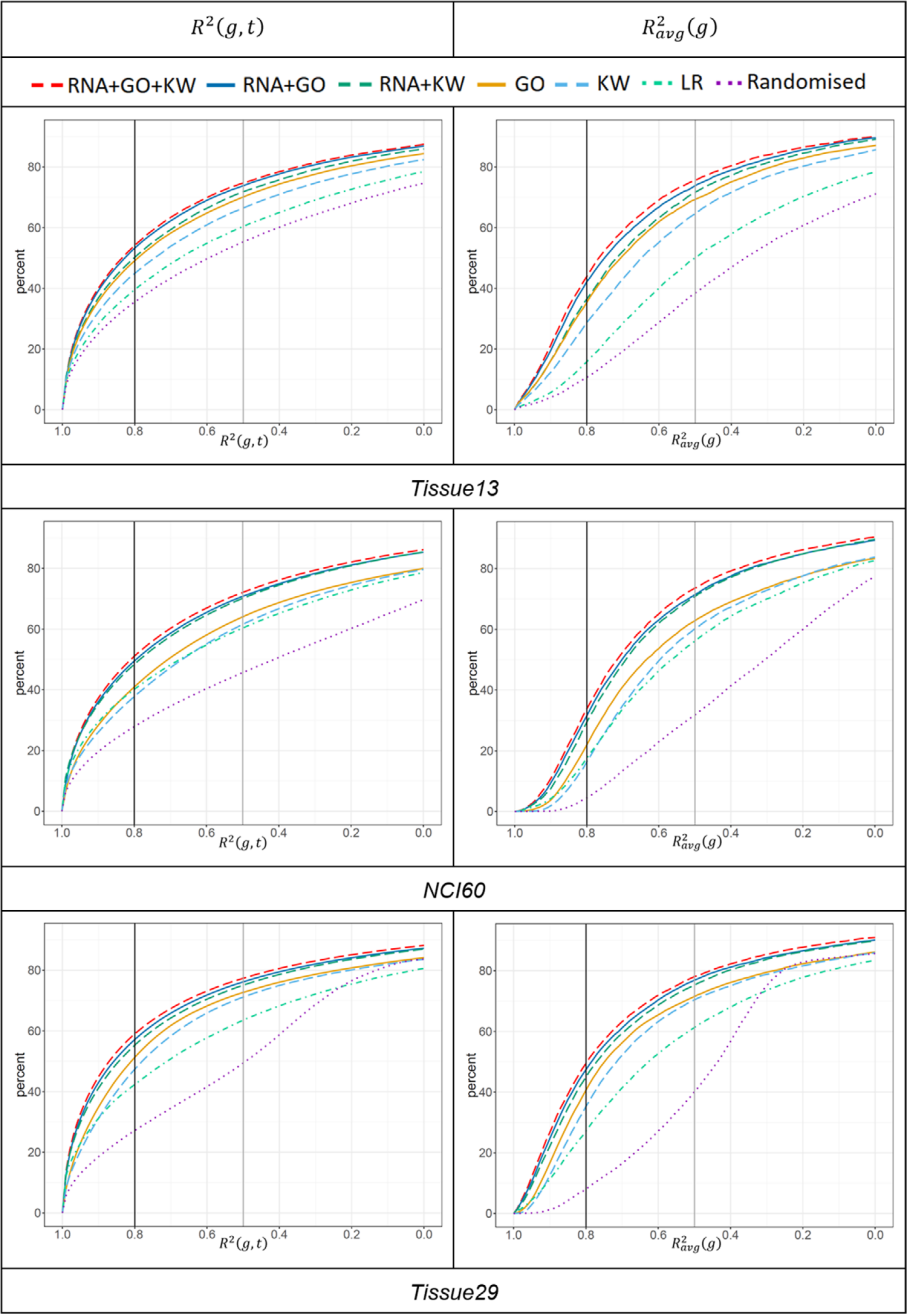
Percentages of genes (on the *y* axes) that can be predicted with $R^{2}$ scores above a certain level (on the *x* axes). Data are shown for all three datasets, seven prediction models, and average scores $R^{2}\left(g,t\right)$ and $R_{\mathrm{avg}}^{2}\left(g\right)$. For briefness, only $R^{2}$ values within the range [0.0,1.0] are shown (i.e., all the curves eventually reach 100% mark, however, at a very low $R^{2}$ values, ranging between −30 and −10).

For the three human datasets, we tested all seven DL network‐based prediction models and compared them to the LR as a benchmark as well as to the Randomised model. As already noted in Experimental Section, the *LR* and *RNA* only models produced almost identical results, therefore the latter is not discussed. The prediction accuracy generated for these datasets is summarized in **Table** **1** and **Figure** **2**.

**Table 1:** The minimal, maximal, and average prediction accuracy for the three human datasets and different prediction models

	Tissue13				NCI60				Tissue29			
Model	$\;R_{\mathrm{Min}}^{2}$	$R_{\mathrm{Max}}^{2}$	$R_{\mathrm{Tavg}}^{2}$	$R_{\mathrm{Davg}}^{2}$	$\;R_{\mathrm{Min}}^{2}$	$R_{\mathrm{Max}}^{2}$	$R_{\mathrm{Tavg}}^{2}$	$R_{\mathrm{Davg}}^{2}$	$\;R_{\mathrm{Min}}^{2}$	$R_{\mathrm{Max}}^{2}$	$R_{\mathrm{Tavg}}^{2}$	$R_{\mathrm{Davg}}^{2}$
LR	0.076	0.256	0.131	0.163	0.157	0.379	0.315	0.319	0.139	0.368	0.242	0.248
KW	0.221	0.310	0.273	0.302	0.163	0.318	0.283	0.287	0.235	0.396	0.347	0.352
GO	0.283	0.382	0.339	0.369	0.148	0.333	0.303	0.307	0.221	0.416	0.371	0.375
RNA+KW	0.359	0.498	0.410	0.433	0.232	0.549	0.488	0.493	0.323	0.585	0.488	0.493
RNA+GO	0.388	0.545	0.444	0.469	0.224	0.543	0.483	0.488	0.321	0.600	0.500	0.505
RNA+GO+KW	0.408	0.557	0.463	0.488	0.236	0.570	0.508	0.514	0.347	0.631	0.538	0.543

The first notable observation is that both KW and GO gene annotations alone provided better predictions of protein abundance than RNA (*LR*) abundances alone. The impact of KW and GO was roughly similar, although in most cases GO performed slightly better. However, despite the fact that RNA on its own was a poorer predictor, the GO or KW predictions improved significantly when they were used together with RNA expression values. These observations were consistent across all three human datasets. The best predictions were obtained by the *RNA+KW+GO* model with $R_{\mathrm{Davg}}^{2}$ values ranging between 0.49 and 0.54 (compared to the *LR* range of 0.16–0.32). At an individual tissue level, the best predictions were obtained for liver. Note that in this tissue, the correlation between protein and RNA abundances were also the highest. A noticeable outlier was bone marrow in the *Tissue29* dataset with $R^{2}=\;0.347$, but for most of the tissues, t score $R^{2}\left[t\right]$ was close to the average value. The same was true for the *NCI60* dataset, which had two cell lines as outliers: HCT116 and X7860, with $R^{2}$ equal to 0.236 and 0.357, respectively. The improvement of the *RNA+KW+GO* model over the *LR* model was higher in the case of the tissue datasets than for the cell line dataset.

The DL network can be trained on a dataset with any number of samples (tissues); however, the number of available samples has very limited impact on prediction accuracy, and even data on a single tissue give similar accuracy as the use of the whole dataset. Regarding the number of data points (proteins) for the datasets tested, it was observed that the prediction accuracy remained practically stable with less than 50% of randomly selected data points removed, and then started to drop significantly, if sizes of data sets were further reduced (e.g., for *Tissue13* dataset with 9637 proteins, the prediction accuracy changes little until the reduced dataset contains at least 5000 proteins, but can start to drop rapidly, if smaller size subsets are chosen).

We also assessed the stability of $R_{\mathrm{Davg}}^{2}$ over different runs (from ten runs in total for each dataset), obtaining standard deviation (SD) values ranging between 0.04 and 0.06 for the different prediction models and datasets, showing that the predictions are stable. The $R^{2}$ values shown here represent averages over these ten test runs. For the complete data of all experimental results covering all tissues and cell lines see Supporting Information.

As one can observe in Figure 2, all the DL models (apart from *Randomised*) clearly outperformed *LR*. For the best prediction model *RNA+GO+KW*, the ratio of gene pairs from all samples that could be predicted with the $R^{2}\left(g,t\right)$ score of at least 0.5 ranged from 72% (*NCI60*) to 77% (*Tissue29*), and from 51% to 59% in the case of the 0.8 score threshold. As a rough approximation, these $R^{2}\left(g,t\right)$ thresholds can be interpreted as correlations of 0.7 and 0.9, respectively.

Note that for $R_{avg}^{2}\left(g\right)$, similar percentages of proteins reached a 0.5 threshold, but percentages decreased for larger thresholds (34% to 49%, for scores of 0.8). Notably, similar behavior patterns persisted for all prediction models and all three human datasets.

**Figure 3:**
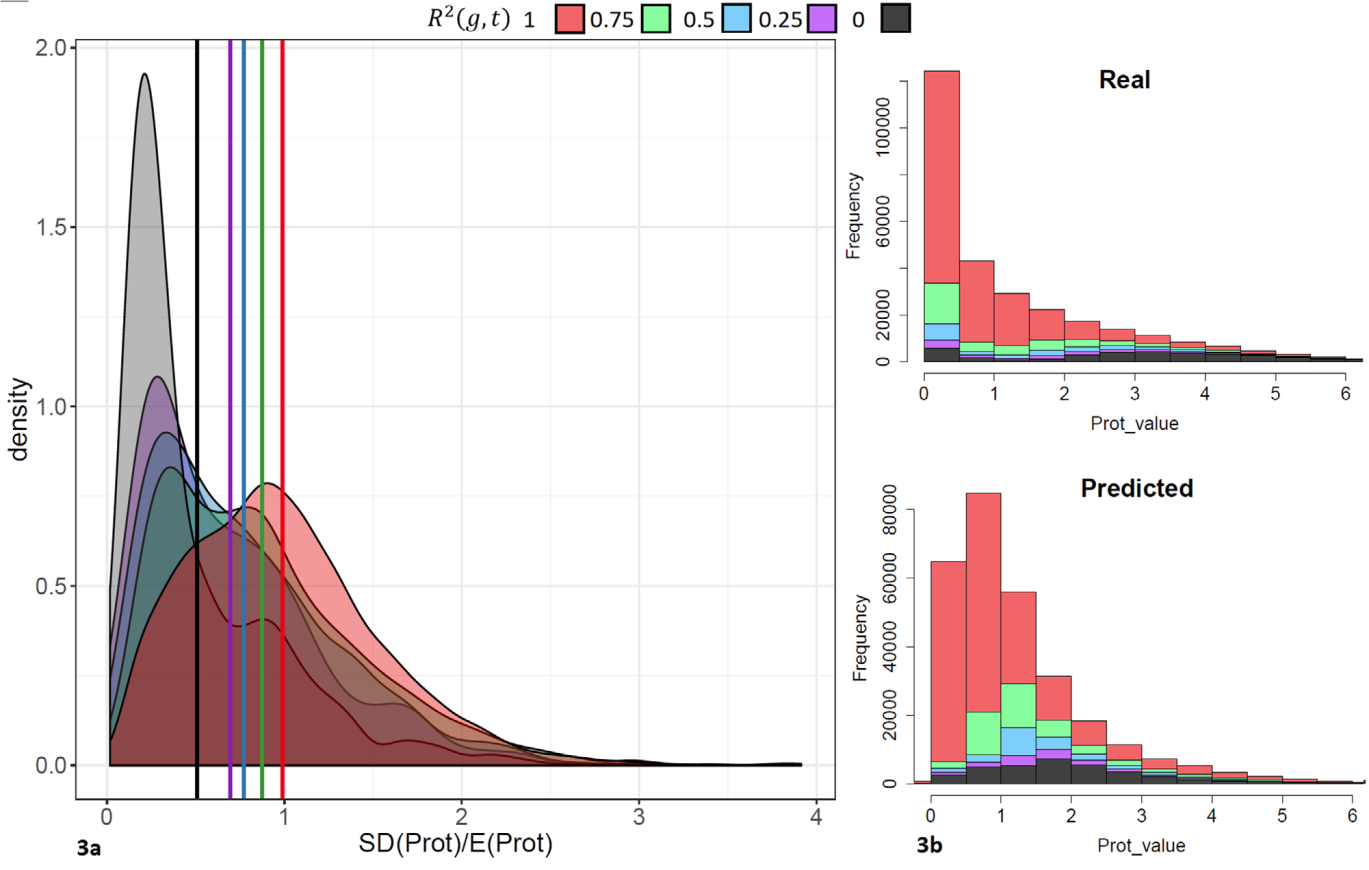
Representation of the relationship between variability of protein concentrations among tissues (SD divided by the mean value) and the prediction accuracy $R^{2}\left(g,t\right)$ (a), and between prediction accuracy and protein abundance (b).

A possible explanation for this is that for different tissues or cell lines, the highest prediction accuracy is achieved on the different set of genes. **Figure** **3** shows the relationship between the density of relative variability of protein concentrations among tissues (SD divided by the mean protein abundance values) and the prediction accuracy $R^{2}\left(g,t\right)$ for the *Tissue29* dataset.

A modest trend could be observed in the results: Better predictions were obtained for proteins with larger abundance variability between different tissues. A possible explanation is that the concentrations of such proteins are more affected by the levels of RNA expression and the accuracy of the experimental quantification. The figure also shows that better predictions were obtained for proteins with lower abundances. Overall, our results show that the abundances of proteins not measured experimentally can be predicted in silico from gene annotations and RNA expression values of the respective genes.

### 3.2 Predictive Value of Specific Annotation Terms

**Figure 4:**
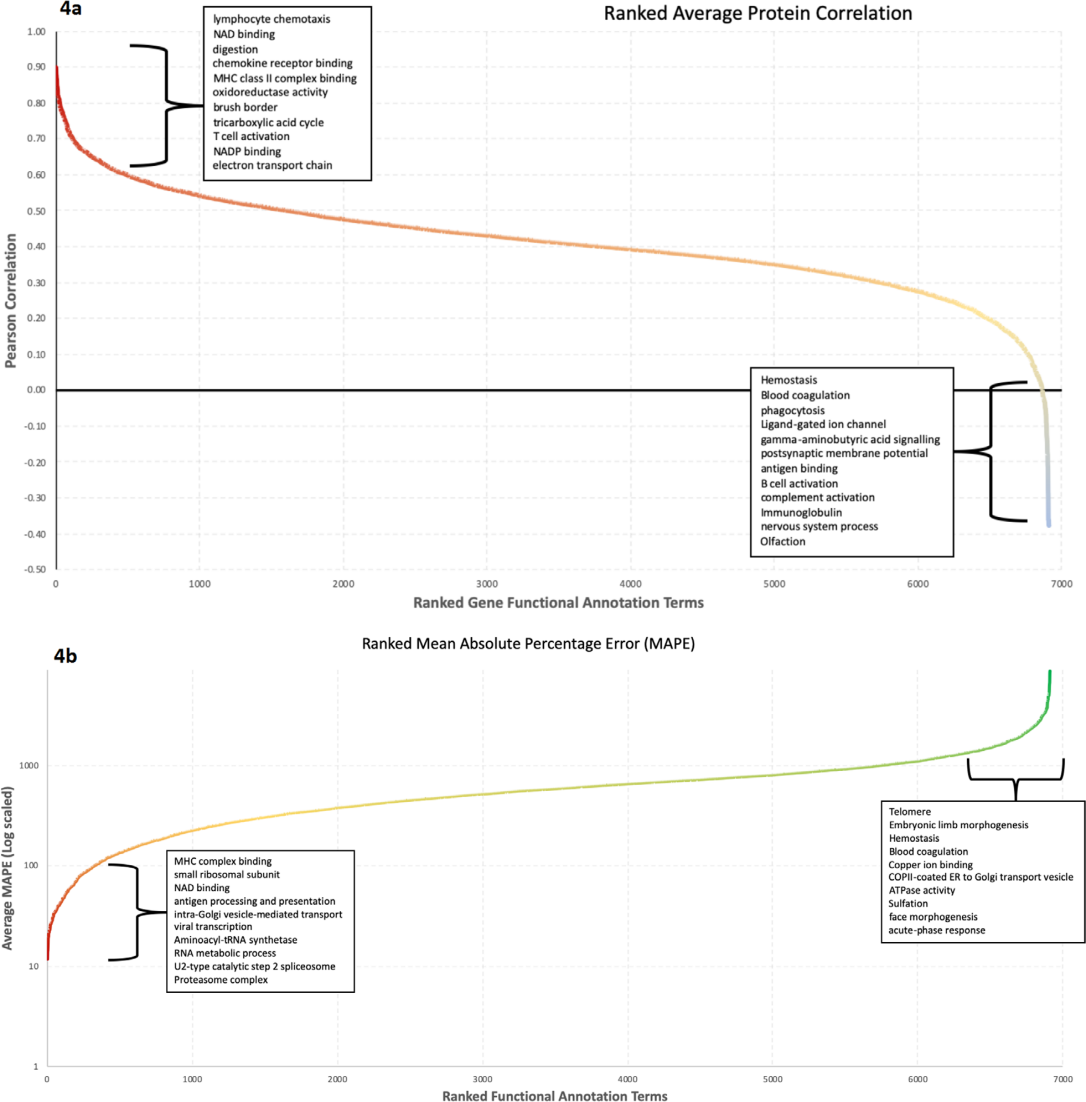
Comparison of the predictive accuracy within the different functional annotation categories. For this analysis, the average Pearson's correlation value between the predicted and observed protein abundances across the 29 human tissues in the Tissue29 dataset was computed. a) The plot displays functional annotation terms ranked by their average gene correlation across the tissues. b) The plot displays the MAPE values for each term. The terms were filtered to only include those ones containing at least five identified proteins that had values for at least 25% of the tissues. The ten best and worst terms in the two analyses (with a minimum of 30 genes represented by the term) are shown on each plot.

Since protein abundance prediction accuracy improved with GO and KW functional annotations, we conclude that this context information plays a role in the estimation. Therefore, we explored if there are particular sub‐categories of genes that can be predicted with greater accuracy than others. Focusing on the *Tissue29* dataset, we examined this in two ways. First, the average correlation for each functional GO or KW category was calculated by comparing the values of each gene across the tissues between the original experimental protein abundances and the predicted values. Second, the mean absolute percentage error (MAPE) of the predicted versus experimental protein expression was calculated for all genes in each functional category. **Figure** **4** shows the GO and KW terms ranked by correlation and MAPE. A wide range in the correlation scores and MAPE values between functional terms were found. The full table of annotational terms and their average correlation and MAPE can be found in Supporting Information. The best correlating terms mostly relate to metabolism and high turn‐over proteins, which are strongly regulated at the transcriptional level. The poorest correlating terms contained proteins which are known to be difficult to experimentally quantify using MS, such as transmembrane proteins. This finding followed the general pattern observed in previous comparisons between the proteome and the transcriptome.^[^ ^9^, ^10^, ^11^ ^]^

We also found some evidence that some high abundance errors and poor correlation values found could in part be attributed to the accuracy of the experimental protein detection and quantification. An interesting example is the “olfaction” term (GO:0004984). These proteins (olfactory receptors, which are present in olfactory receptor neurons and are responsible for the sense of smell) are notoriously difficult to detect and due to their well‐known tissue specificity, it is highly likely that these represent experimental false‐positive identifications rather than poor predictions.^[^ ^24^ ^]^ When more stringent peptide confidence measures were applied, as it was the case in the original analysis of this dataset, these proteins were removed from the dataset. The high error rate can be attributed to the fact that the predicted abundance is very low (less than 0.1, suggesting the protein is not present in these tissues), whereas the experimental values were significantly higher and probably incorrect. Another category of proteins showing poor correlation are serum proteins, which suggests contamination by blood in some experimental tissues.

### 3.3 Model Transferability to Mouse

**Figure 5:**
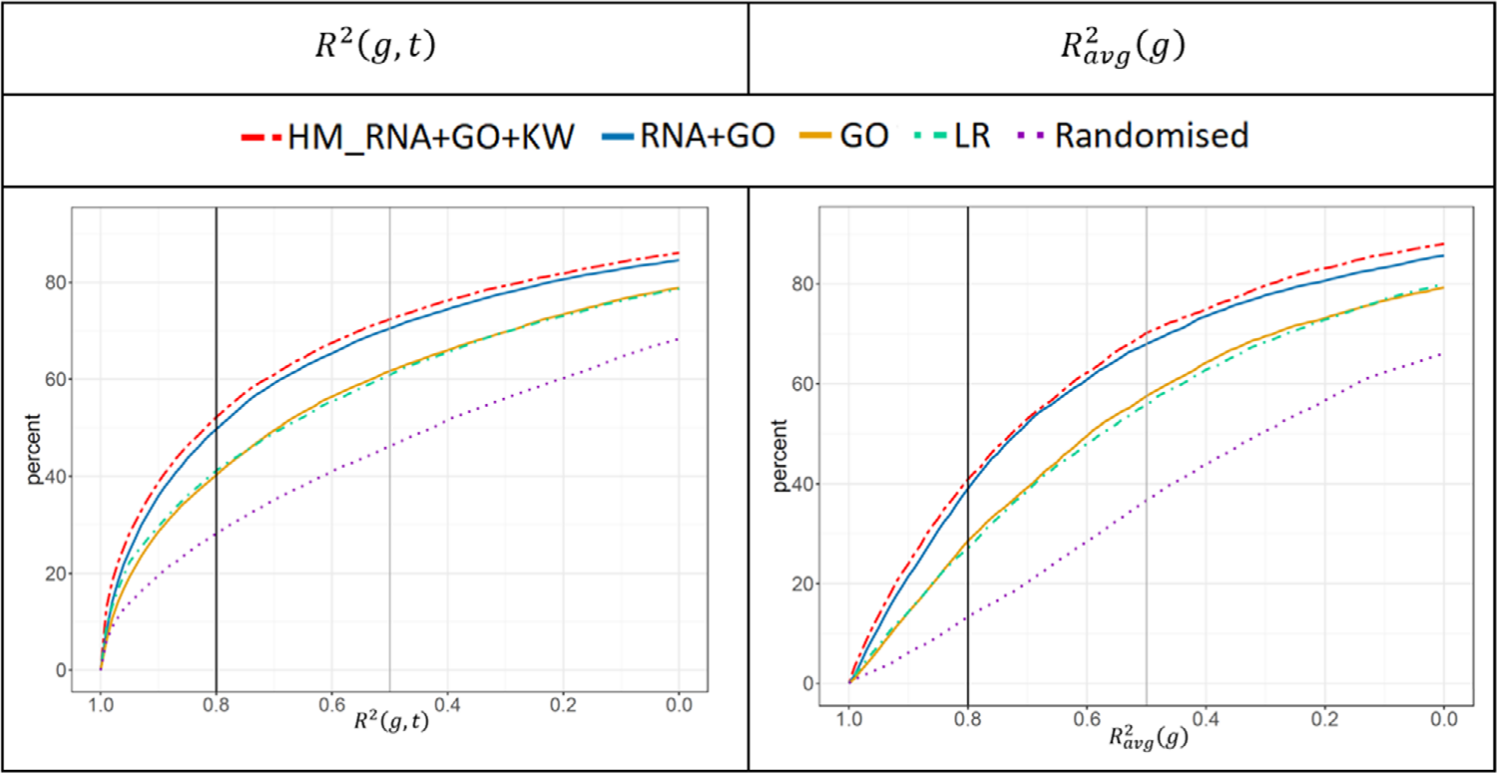
Percentages of mouse genes (on the *y* axes) that can be predicted above $R^{2}$ thresholds (on the *x* axes) within the range [0.0,1.0] using $R^{2}\left(g,t\right)$ and $R_{\mathrm{avg}}^{2}\left(g\right)$ scores. Data are shown for four prediction models as well as for the HM_RNA+GO+KW “model”, which applied coefficients computed from the human Tissue29 dataset to predict abundance of homologous mouse proteins on the basis of RNA expression data alone.

Next, our aim was to test whether the models were transferable from human to a different species. In the case of the mouse dataset *MouseTissue3*, four DL models were tested. The results are summarized in **Table** **2** and **Figure** **5**. The results were similar to those found in human data with $R_{\mathrm{Davg}}^{2}=\;0.45$ for the *RNA+GO* model in comparison to a 0.3 value obtained for the *LR* model. The percentages of genes predictable with scores above 0.5 and 0.8 were 70% and 50%, respectively (62% and 40% for *LR*). Nevertheless, predictions arising from GO annotations only gave results similar to *LR*, which could be explained by the sparser GO annotations available for mouse.

**Table 2:** The minimal, maximal, and average prediction accuracy for mouse datasets coming from mouse gene expression values and/or GO annotations, and from the coefficients assigned to mouse genes by training DL prediction models on the human Tissue29 dataset

Model	$\;R_{\mathrm{Min}}^{2}$	$R_{\mathrm{Max}}^{2}$	$R_{\mathrm{Tavg}}^{2}$	$R_{\mathrm{Davg}}^{2}$
LR	0.208	0.385	0.304	0.306
GO	0.194	0.337	0.262	0.264
RNA+GO	0.338	0.546	0.450	0.451
HM_RNA+KW	0.415	0.551	0.475	0.473
HM_RNA+GO	0.424	0.548	0.494	0.492
HM_RNA+GO+KW	0.445	0.577	0.515	0.513

We used the α(g) and β(g)coefficients assigned to genes by DL network trained on human data (specifically for the human *Tissue29* dataset for two tissues—liver and testis) and applied these to homologous genes in mouse tissues. Such homologues were available for 5388 of 6591 mouse genes measured in the *MouseTissue3* dataset.

Unexpectedly, we observed that the coefficient computed by the *RNA+GO* model trained on human data provided better predictions than the same model trained on the mouse dataset, indicating that models derived from larger sets with more comprehensive GO annotations appear to be applicable to homologous genes. No‐tissue specificity of the gene specific coefficients was observed. The best predictions were obtained from coefficients computed by the DL network without including tissue information. In principle, this is consistent with observations from related work where such coefficients were assigned directly from the measurements.^[^ ^25^, ^26^ ^]^ However, given that we used data from only two common tissues, this does not allow us to reach reliable conclusions. Tissue‐specificity of expression of some genes has been used to derive marker sets.^[^ ^27^ ^]^ The challenge now is how such tissue‐related information can be extracted from available gene annotation data.

## 4 Discussion

Deep learning approaches have recently been applied to predict gene expression levels in a different context;^[^ ^28^ ^]^ nevertheless to the best of our knowledge, existing approaches for protein abundance prediction focus on imputing partially missing values or as is the case with the NCI‐CPTAC DREAM proteogenomics challenge, to infer proteomes in new samples from genomics datasets. Our method is aimed at extrapolating the measured experimental values to unobserved proteins (Figure 1). One previous protein abundance prediction approach used linear regression applied to RNA levels measured for 512 genes in Daoy medulloblastoma cells.^[^ ^29^ ^]^ The authors reported accuracy $R^{2}=\;0.29$. A higher prediction accuracy $R^{2}=\;0.41$ was reported in a dataset of 5279 genes in the NIH3T3 mouse fibroblast cell line.^[^ ^30^ ^]^ The predictions here were based on gene‐specific coefficients that were assigned based on measurements of translation and degradation rates at three different timepoints. The authors also reported that these rates were similar for homologous genes, which is consistent with our observation that models trained on human data can be applied to homologous mouse genes. In comparison, the $R^{2}$ values that we obtained using the RNA+GO+KW model for all the four datasets exceeded the reported $R^{2}=\;0.41$; moreover, our assignment of gene specific coefficients are obtained purely in silico from GO and KW annotations rather than from custom‐designed lab experiments. Another study involving 9 human cell lines and 11 tissues^[^ ^25^ ^]^ assigned gene‐specific coefficients based on RNA‐to‐protein conversion rates. This approach achieved a high prediction accuracy $R^{2}$ of up to 0.8, but only for a small set of 55 “hand‐picked” genes. In such cases of tailored small sets, higher accuracy should be expected.

Recently, an NCI‐CPTAC DREAM proteogenomics challenge has focused on the prediction of protein expression levels across samples from cancer patients, using genomics data combined with protein annotational information.^[^ ^12^, ^13^ ^]^ Various predictive linear and non‐linear models were evaluated using isobaric labeled (iTRAQ) proteomics and RNAseq datasets generated by CPTAC. These models attempt to infer protein expression samples based on the transcript abundances supplemented with protein features such as interactions and conservation attributes. Of the various approaches they examined, the best method was an ensemble approach using a random forest algorithm and achieving an *R* score of 0.53, which is comparable to what we achieve for the prediction of unobserved proteins.^[^ ^13^ ^]^

As noted, our approach is aimed at extrapolating label‐free global protein expression values to the full proteome, including the experimentally unobserved proteins, and thus our results are not directly comparable with these existing methods.^[^ ^31^ ^]^ Nevertheless, we can achieve the accuracy of $R^{2}\;$ scores between 0.46 and 0.54.

In our study, we have solely applied our method to label‐free MS proteomics datasets that used MS1 intensity‐based quantification (iBAQ). This particular type of experimental protein expression data was selected because currently it is the most widely used approach for protein expression data in public databases. We have opted to use datasets analyzed using MaxQuant, as it reflects a prominent workflow in label‐free proteomics. Obviously, our predictions of the true protein abundances are only as good as the data for the experimentally measured proteins. There are a myriad of methods and tools for assessing the true protein abundance in proteomics experiments; however, there are limited ways to assess the accuracy of protein quantification on a large scale without using synthetic or purified protein standards. Although some benchmarking proteomics datasets have been generated, the complication for using them in our approach is that data are needed with proteins behaving in context of their biological annotations, and with a suitable paired RNAseq expression dataset. To the best of our knowledge, such a benchmarking dataset does not yet exist. As it is essential for our method that meaningful protein annotations and complementary RNA expression data are available, the application of our method to peptide‐level quantifications would be problematic.

In theory, since the neural network is trained on each dataset independently, our method could potentially be extended to support other experimental methodologies for protein quantification such as labeled MS approaches and absolute quantification experiments. However, further testing and optimization would likely be needed in these cases, which are beyond the scope of this study.

In most applications of our method, an independent model will be trained for each dataset for which values are to be predicted. However, we have examined the possibility of using a model trained on one dataset to predict values in a different experiment and demonstrated that our DL method provides a good predictive model across datasets, even across different species, such as human and mouse (exploiting gene homology mappings). In fact, training the DL on larger more comprehensive datasets and applying it to a smaller dataset can yield better accuracy in the prediction. For instance, the model trained on a larger human dataset transferred to mouse gave a better prediction of values than the model trained on a small mouse dataset. It has to be noted that the prediction accuracy varies between proteins; moreover, the particularly difficult‐to‐predict proteins depended on the biological sample. Nevertheless, the prediction models were relatively stable across datasets and a model learned from one dataset could be applied to predict unobserved proteins in another dataset.

It may seem surprising that GO or KW annotations are better predictors of the unobserved protein abundances than the RNA measurements. A possible explanation for this is that gene annotation terms provide links between proteins that have related functions, are parts of the same protein complexes, or are co‐regulated and thus are present in similar samples in similar abundances. It is important to note, however, that when combined with information coming from gene annotations, RNA measurements improved the predictions accuracy significantly.

We have investigated the performance of the method for different protein subsets by looking at the correlation of predicted protein values to experimental protein values across tissues, taking the average correlation for each annotational term. Additionally, we calculated the average MAPE for proteins in each subset. These subsets of proteins showed a range of accuracies between the different functional annotational terms. There appear to be two factors driving the extremes in accuracy between terms. The best correlating and most accurately predicted proteins appear to include annotational terms, such as metabolism and immune response. Such proteins are known to be strongly regulated at the transcriptional level.^[^ ^11^ ^]^ This is explained by the fact that the predictive power of the RNAseq values is at its best for these categories and is significantly boosting the prediction accuracy for these proteins.

For the least accurate functional categories, there seems to be a different explanation. These terms generally reflect categories of proteins that would correlate very poorly with RNAseq values. These include tissue‐specific protein sets, potentially proteins coming from contaminations in the sample, or proteins that are difficult to detect by experimental MS approaches. Additionally, one key example we can note is olfaction‐related proteins, which are notoriously difficult to identify via MS and have very high tissue specificity. However, olfactory receptors have been identified in the datasets used here. These are highly likely to be false positives, especially since these identifications are not present in the original analyses of these experiments, where stricter peptide confidence filtering was applied. These proteins display very low accuracy between predicted and experimental values. In this sense, the predicted expression is more realistic than the observed experimental values. This demonstrates the power of our method for identifying aberrant protein expression where some confounding factors, such as false‐positive identifications, variant proteins, and protein modifications, are impacting the experimental quantification. Due to the nature of the predictive method we are applying, a protein will always be given a predicted expression value, and hence there will always be a background noise even if the protein is not biologically present in that sample. This is worth noting when the predictions are applied to truly unobserved proteins, and a minimum predicted expression threshold should therefore be imposed.

To test our predictions, we excluded 10% of the proteins in each round of training and then built 100 different models for each dataset and only looked at the predictive accuracy of proteins masked from the training set. This was done to enable the investigation of the accuracy of predicted proteins unobserved in all samples, extending the proteome coverage, based on the RNAseq and the functional annotational relationships to the rest of the proteomics experimental data. However, this approach is not suitable for examining the accuracy of the imputation for partially missing proteins. Although the model can be applied for this purpose, we have not explored this application here and can only hypothesize that performance would not necessarily be better than existing approaches. To take full advantage of the deep learning methodology for imputation, further optimization and testing would also be required.

The fact that a model trained using the human data predicted mouse proteins better than using the model trained on the mouse model demonstrates that the accuracy of the predictions can be improved by having a larger input dataset with more complete functional annotation. To assess the accuracies of any given model when applying the method to new datasets, a similar cross validation strategy should be implemented, leaving a fraction of the data out of each model over multiple iterations. This should provide a good indication for the overall accuracy for unobserved proteins in any particular functional category.

Given that our protein abundance measurement predictions are tested by masking a subset of the proteins that actually are measured experimentally, the question as to what extent our conclusions could be reliably transferred on the truly unobserved proteins can arise. Is it possible that the truly unobserved proteins have properties different from those of the measured ones and therefore our computational predictions are not reliable? This possibility is difficult to test directly; however, the observation that our models can be transferred between datasets with different sets of proteins absent in different experiments provides indirect evidence that our predictions do apply to all proteins. We also highlight that in the assessment of the accuracy of annotations, the predicted values can actually be used to expose aberrant protein abundance values (i.e., in the case of the olfactory receptors) and situations where a false identification or quantification has occurred in the experimental data.

In our computational study to train DL networks, we specifically use GO or UniProt KW as contextual information; in principle, our method allows for using any context information that can be associated with genes, for instance, protein sequence, domain features, and/ or KEGG pathways, among others. Taken together, this study demonstrates that our method can be used to estimate the abundance for proteins that are unobserved in label‐free MS experiments and to reveal instances where measurements may not be reliable. This provides greater coverage of pathways and protein networks for improved downstream analysis. Additionally, it can highlight unobserved or aberrantly expressed proteins with biological relevance that can be targeted in further experiments.

## 5 Associated Data

The datasets used for the study are obtained from the following Expression Atlas datasets: *Tissue13* from E‐PROT‐1 (coming from PRIDE datasets PXD000561 and PXD002967) and E‐MTAB‐2836 (RNA expression); *NCI60* from E‐PROT‐25 (PRIDE dataset PXD005940) and E‐MTAB‐2770 (RNA expression); *Tissue29* from E‐PROT‐29 (PRIDE dataset PXD010154) and E‐MTAB‐2836 (RNA expression); *MouseTissue3* from E‐PROT‐13 and E‐GEOD‐43721 (RNA expression). Pre‐processed datasets and the developed software are available at GitHub: https://github.com/IMCS-Bioinformatics/DLNetworkForProteinAbundancePrediction. More details about datasets and software are provided in Supporting Information.

## Conflict of Interest

The authors declare no conflict of interest.

## Supporting information

Supporting Information

Supporting Information

Supporting Information

Supporting Information

Supporting Information

Supporting Information

Supporting Information

Supporting Information

Supporting Information

## References

[1] N. C. van de Merbel , Bioanalysis 2019, 11, 629. 30983409 10.4155/bio-2019-0032

[2] B. C. Collins , R. Aebersold , Nat. Biotechnol. 2018, 36, 1051. 30412198 10.1038/nbt.4288

[3] R. A. Zubarev , Proteomics 2013, 13, 723. 23307342 10.1002/pmic.201200451

[4] C. Lazar , L. Gatto , M. Ferro , C. Bruley , T. Burger , J. Proteome Res. 2016, 15, 1116. 26906401 10.1021/acs.jproteome.5b00981

[5] B. J. M. Webb‐Robertson , H. K. Wiberg , M. M. Matzke , J. N. Brown , J. Wang , J. E. McDermott , R. D. Smith , K. D. Rodland , T. O. Metz , J. G. Pounds , K. M. Waters , J. Proteome Res. 2015, 14, 1993. 25855118 10.1021/pr501138h PMC4776766

[6] Y. S. Liu , A. Beyer , R. Aebersold , Cell 2016, 165, 535. 27104977 10.1016/j.cell.2016.03.014

[7] S. Marguerat , A. Schmidt , S. Codlin , W. Chen , R. Aebersold , J. Bahler , Cell 2012, 151, 671. 23101633 10.1016/j.cell.2012.09.019 PMC3482660

[8] A. L. Bauernfeind , C. C. Babbitt , BMC Genomics 2017, 18. 10.1186/s12864-017-3674-x PMC5402646 28438116

[9] M. Jovanovic , M. S. Rooney , P. Mertins , D. Przybylski , N. Chevrier , R. Satija , E. H. Rodriguez , A. P. Fields , S. Schwartz , R. Raychowdhury , M. R. Mumbach , T. Eisenhaure , M. Rabani , D. Gennert , D. N. Lu , T. Delorey , J. S. Weissman , S. A. Carr , N. Hacohen , A. Regev , Science 2015, 347, 1259038. 25745177 10.1126/science.1259038 PMC4506746

[10] Z. Cheng , G. S. Teo , S. Krueger , T. M. Rock , H. W. L. Koh , H. Choi , C. Vogel , Mol. Sys. Biol. 2016, 12, 855. 10.15252/msb.20156423 PMC4731011 26792871

[11] T. I. Roumeliotis , S. P. Williams , E. Goncalves , C. Alsinet , M. D. Velasco‐Herrera , N. Aben , F. Z. Ghavidel , M. Michaut , M. Schubert , S. Price , J. C. Wright , L. Yu , M. Yang , R. Dienstmann , J. Guinney , P. Beltrao , A. Brazma , M. Pardo , O. Stegle , D. J. Adams , L. Wessels , J. Saez‐Rodriguez , U. McDermott , J. S. Choudhary , Cell Rep. 2017, 20, 2201. 28854368 10.1016/j.celrep.2017.08.010 PMC5583477

[12] M. Yang , F. Petralia , Z. Li , H. Li , W. Ma , X. Song , S. Kim , H. Lee , H. Yu , B. Lee , S. Bae , E. Heo , J. Kaczmarczyk , P. Stępniak , M. Warchoł , T. Yu , A. P. Calinawan , P. C. Boutros , S. H. Payne , B. Reva , E. Boja , H. Rodriguez , G. Stolovitzky , Y. Guan , J. Kang , P. Wang , D. Fenyö , J. Saez‐Rodriguez , Cell Sys. 2020, 11, P186.

[13] H. Y. Li , O. Siddiqui , H. J. Zhang , Y. F. Guan , BMC Biol. 2019, 17, 14. 31870366 10.1186/s12915-019-0730-9 PMC6929375

[14] M. S. Kim , S. M. Pinto , D. Getnet , R. S. Nirujogi , S. S. Manda , R. Chaerkady , A. K. Madugundu , D. S. Kelkar , R. Isserlin , S. Jain , J. K. Thomas , B. Muthusamy , P. Leal‐Rojas , P. Kumar , N. A. Sahasrabuddhe , L. Balakrishnan , J. Advani , B. George , S. Renuse , L. D. N. Selvan , A. H. Patil , V. Nanjappa , A. Radhakrishnan , S. Prasad , T. Subbannayya , R. Raju , M. Kumar , S. K. Sreenivasamurthy , A. Marimuthu , G. J. Sathe , et al., Nature 2014, 509, 575. 24870542 10.1038/nature13302 PMC4403737

[15] J. C. Wright , J. Mudge , H. Weisser , M. P. Barzine , J. M. Gonzalez , A. Brazma , J. S. Choudhary , J. Harrow , Nat. Commun. 2016, 7, 11778. 27250503 10.1038/ncomms11778 PMC4895710

[16] A. M. Gholami , H. Hahne , Z. X. Wu , F. J. Auer , C. Meng , M. Wilhelm , B. Kuster , Cell Rep. 2013, 4, 609. 23933261 10.1016/j.celrep.2013.07.018

[17] D. X. Wang , B. Eraslan , T. Wieland , B. Hallstrom , T. Hopf , D. P. Zolg , J. Zecha , A. Asplund , L. H. Li , C. Meng , M. Frejno , T. Schmidt , K. Schnatbaum , M. Wilhelm , F. Ponten , M. Uhlen , J. Gagneur , H. Hahne , B. Kuster , Mol. Sys. Biol. 2019, 15, e8503. 10.15252/msb.20188503 PMC6379049 30777892

[18] E. L. Huttlin , M. P. Jedrychowski , J. E. Elias , T. Goswami , R. Rad , S. A. Beausoleil , J. Villen , W. Haas , M. E. Sowa , S. P. Gygi , Cell 2010, 143, 1174. 21183079 10.1016/j.cell.2010.12.001 PMC3035969

[19] I. Papatheodorou , P. Moreno , J. Manning , A. M. P. Fuentes , N. George , S. Fexova , N. A. Fonseca , A. Füllgrabe , M. Green , N. Huang , L. Huerta , H. Iqbal , M. Jianu , S. Mohammed , L. Zhao , A. F. Jarnuczak , S. Jupp , J. Marioni , K. Meyer , R. Petryszak , C. A. Prada Medina , C. Talavera‐López , S. Teichmann , J. A. Vizcaino , A. Brazma , Nucleic Acids Res. 2020, 48, D77. 31665515 10.1093/nar/gkz947 PMC7145605

[20] Y. Perez‐Riverol , A. Csordas , J. W. Bai , M. Bernal‐Llinares , S. Hewapathirana , D. J. Kundu , A. Inuganti , J. Griss , G. Mayer , M. Eisenacher , E. Perez , J. Uszkoreit , J. Pfeuffer , T. Sachsenberg , S. Yilmaz , S. Tiwary , J. Cox , E. Audain , M. Walzer , A. F. Jarnuczak , T. Ternent , A. Brazma , J. A. Vizcaino , Nucleic Acids Res. 2019, 47, D442. 30395289 10.1093/nar/gky1106 PMC6323896

[21] M. Uhlen , L. Fagerberg , B. M. Hallstrom , C. Lindskog , P. Oksvold , A. Mardinoglu , A. Sivertsson , C. Kampf , E. Sjostedt , A. Asplund , I. Olsson , K. Edlund , E. Lundberg , S. Navani , C. A. Szigyarto , J. Odeberg , D. Djureinovic , J. O. Takanen , S. Hober , T. Alm , P. H. Edqvist , H. Berling , H. Tegel , J. Mulder , J. Rockberg , P. Nilsson , J. M. Schwenk , M. Hamsten , K. von Feilitzen , M. Forsberg , et al., Science 2015, 347, 1260419. 25613900 10.1126/science.1260419

[22] M. Soumillon , A. Necsulea , M. Weier , D. Brawand , X. L. Zhang , H. C. Gu , P. Barthes , M. Kokkinaki , S. Nef , A. Gnirke , M. Dym , B. de Massy , T. S. Mikkelsen , H. Kaessmann , Cell Rep. 2013, 3, 2179. 23791531 10.1016/j.celrep.2013.05.031

[23] J. Barretina , G. Caponigro , N. Stransky , K. Venkatesan , A. A. Margolin , S. Kim , C. J. Wilson , J. Lehar , G. V. Kryukov , D. Sonkin , A. Reddy , M. W. Liu , L. Murray , M. F. Berger , J. E. Monahan , P. Morais , J. Meltzer , A. Korejwa , J. Jane‐Valbuena , F. A. Mapa , J. Thibault , E. Bric‐Furlong , P. Raman , A. Shipway , I. H. Engels , J. Cheng , G. Y. K. Yu , J. J. Yu , P. Aspesi , M. de Silva , et al., Nature 2012, 483, 603. 22460905 10.1038/nature11003 PMC3320027

[24] I. Ezkurdia , J. Vazquez , A. Valencia , M. Tress , J. Proteome Res. 2014, 13, 3854. 25014353 10.1021/pr500572z PMC4334283

[25] F. Edfors , F. Danielsson , B. M. Hallstrom , L. Kall , E. Lundberg , F. Ponten , B. Forsstrom , M. Uhlen , Mol. Sys. Biol. 883, 2016, 12. 10.15252/msb.20167144 PMC5081484 27951527

[26] N. Fortelny , C. M. Overall , P. Pavlidis , G. V. C. Freue , Nature 2017, 547, E19. 28748932 10.1038/nature22293

[27] B. Eraslan , D. X. Wang , M. Gusic , H. Prokisch , B. M. Hallstrom , M. Uhlen , A. Asplund , F. Ponten , T. Wieland , T. Hopf , H. Hahne , B. Kuster , J. Gagneur , Mol. Sys. Biol. 2019, 15, e8513. 10.15252/msb.20188513 PMC6379048 30777893

[28] D. R. Kelley , Y. A. Reshef , M. Bileschi , D. Belanger , C. Y. McLean , J. Snoek , Genome Res. 2018, 28, 739. 29588361 10.1101/gr.227819.117 PMC5932613

[29] C. Vogel , R. D. Abreu , D. J. Ko , S. Y. Le , B. A. Shapiro , S. C. Burns , D. Sandhu , D. R. Boutz , E. M. Marcotte , L. O. Penalva , Mol. Sys. Biol. 2010, 6, 400. 10.1038/msb.2010.59 PMC2947365 20739923

[30] B. Schwanhausser , D. Busse , N. Li , G. Dittmar , J. Schuchhardt , J. Wolf , W. Chen , M. Selbach , Nature 2011, 473, 337. 21593866 10.1038/nature10098

[31] M. Wilhelm , J. Schlegl , H. Hahne , A. M. Gholami , M. Lieberenz , M. M. Savitski , E. Ziegler , L. Butzmann , S. Gessulat , H. Marx , T. Mathieson , S. Lemeer , K. Schnatbaum , U. Reimer , H. Wenschuh , M. Mollenhauer , J. Slotta‐Huspenina , J. H. Boese , M. Bantscheff , A. Gerstmair , F. Faerber , B. Kuster , Nature 2014, 509, 582. 24870543 10.1038/nature13319

