# Supplementary figures and images for: Using Deep Learning to Extrapolate Protein Expression Measurements
Source: Proteomics. 2020 Oct 16;20(21-22):2000009. doi: 10.1002/pmic.202000009 (PMC7757209; doi:10.1002/pmic.202000009)

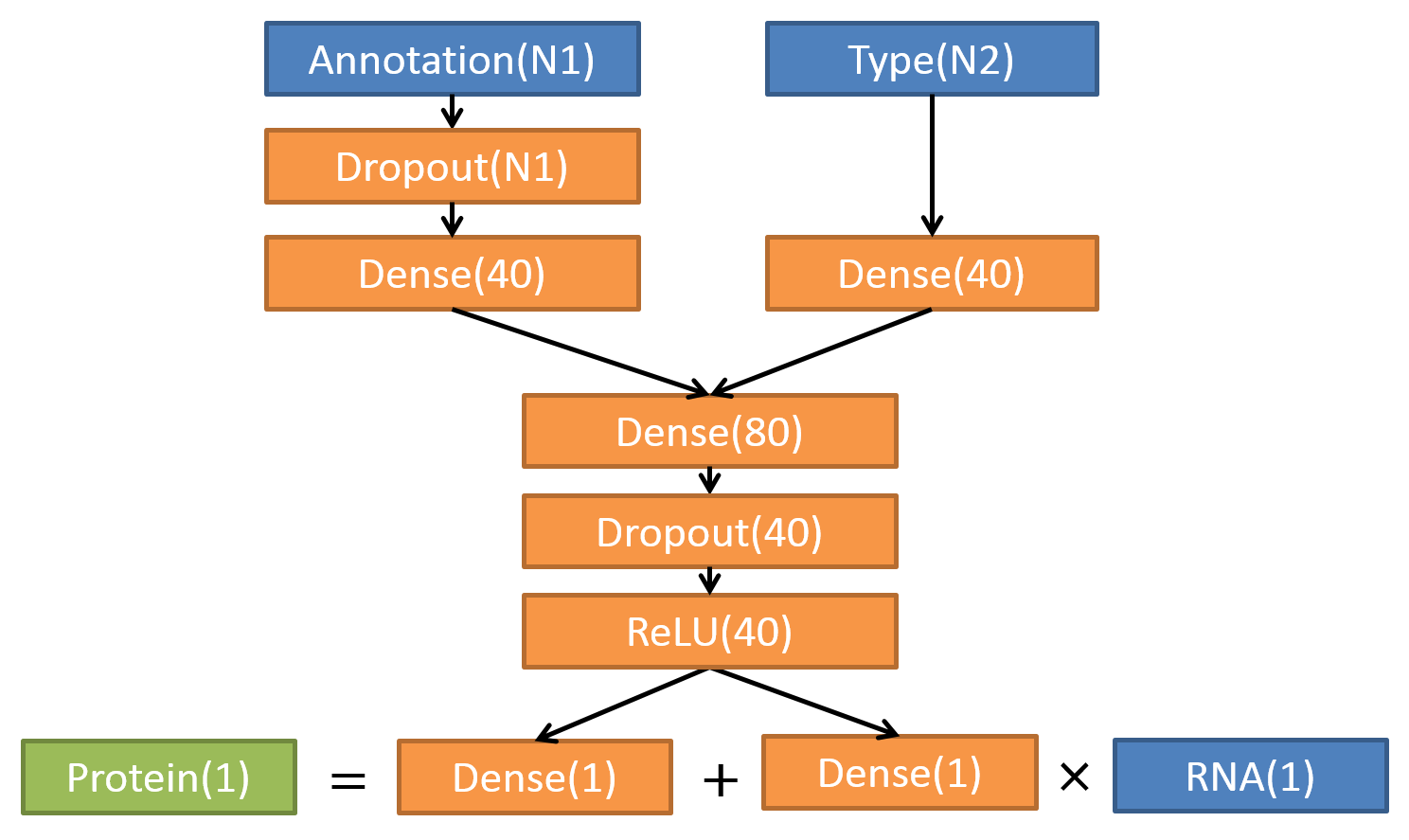

Supplement: Supplementary file 1 — Supporting Information [file PMIC-20-2000009-s009.tif]

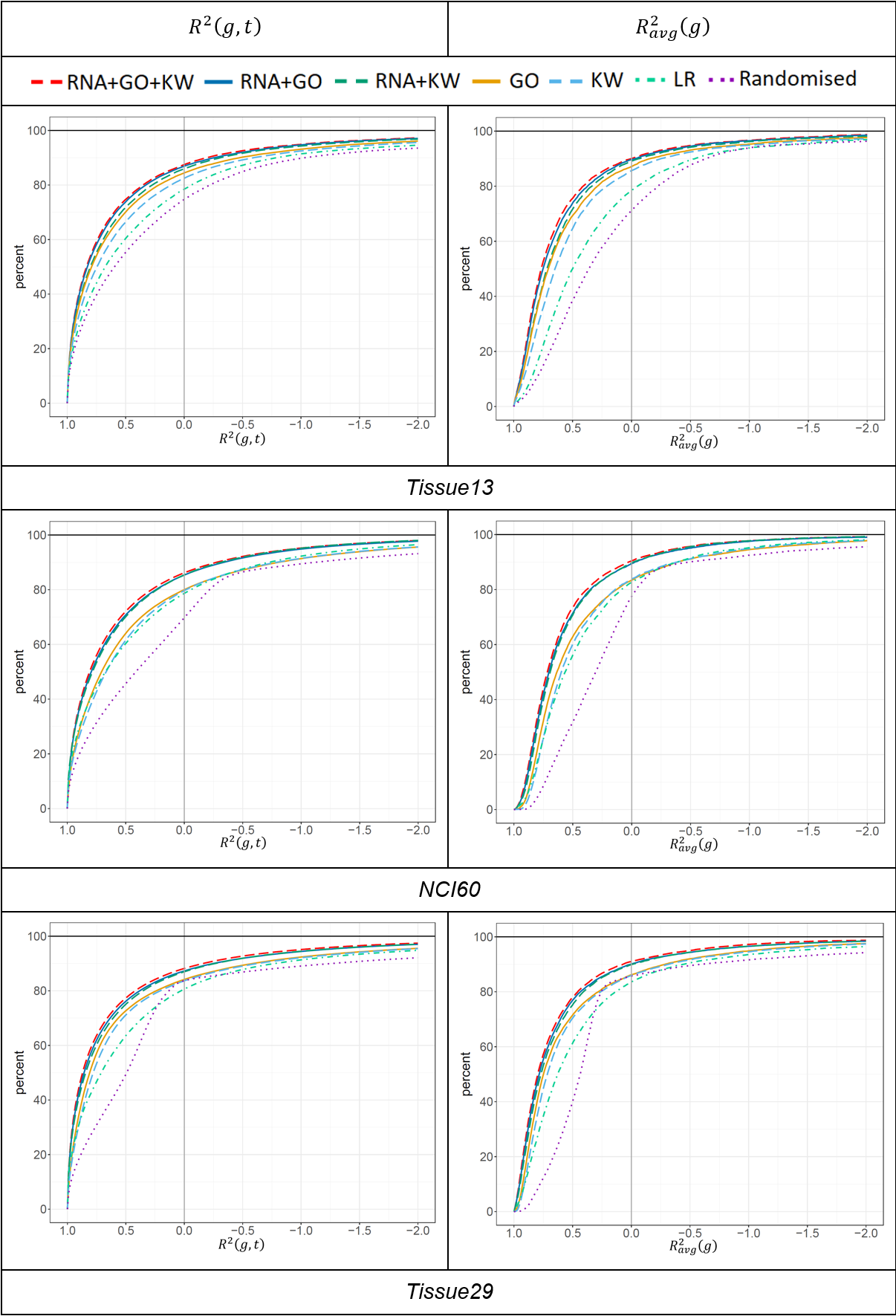

Supplement: Supplementary file 2 — Supporting Information [file PMIC-20-2000009-s001.tif]

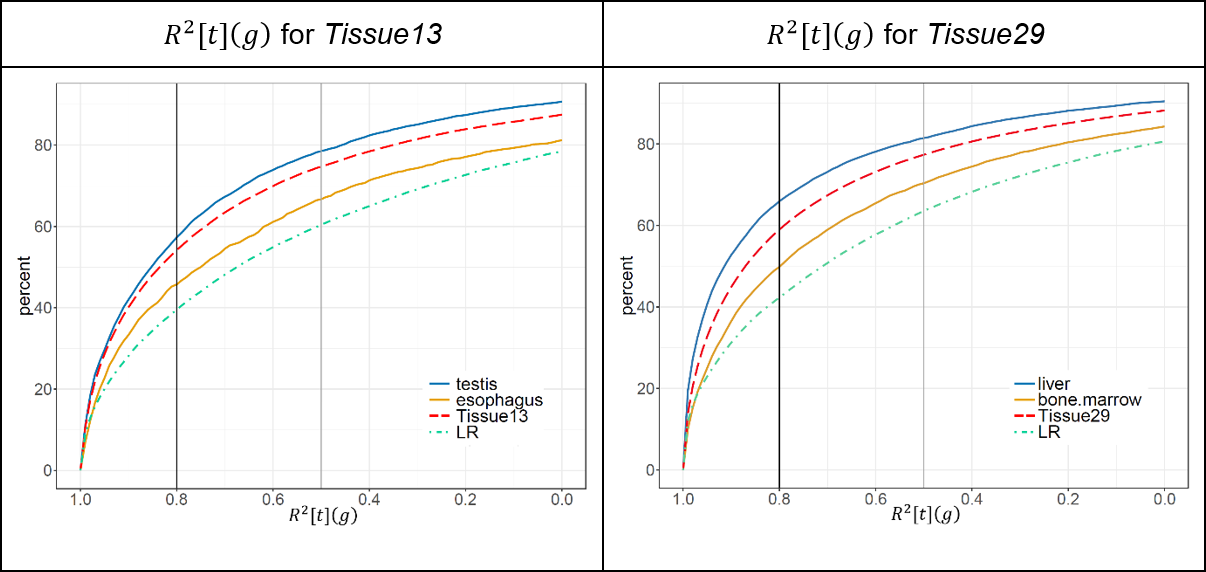

Supplement: Supplementary file 3 — Supporting Information [file PMIC-20-2000009-s002.tif]

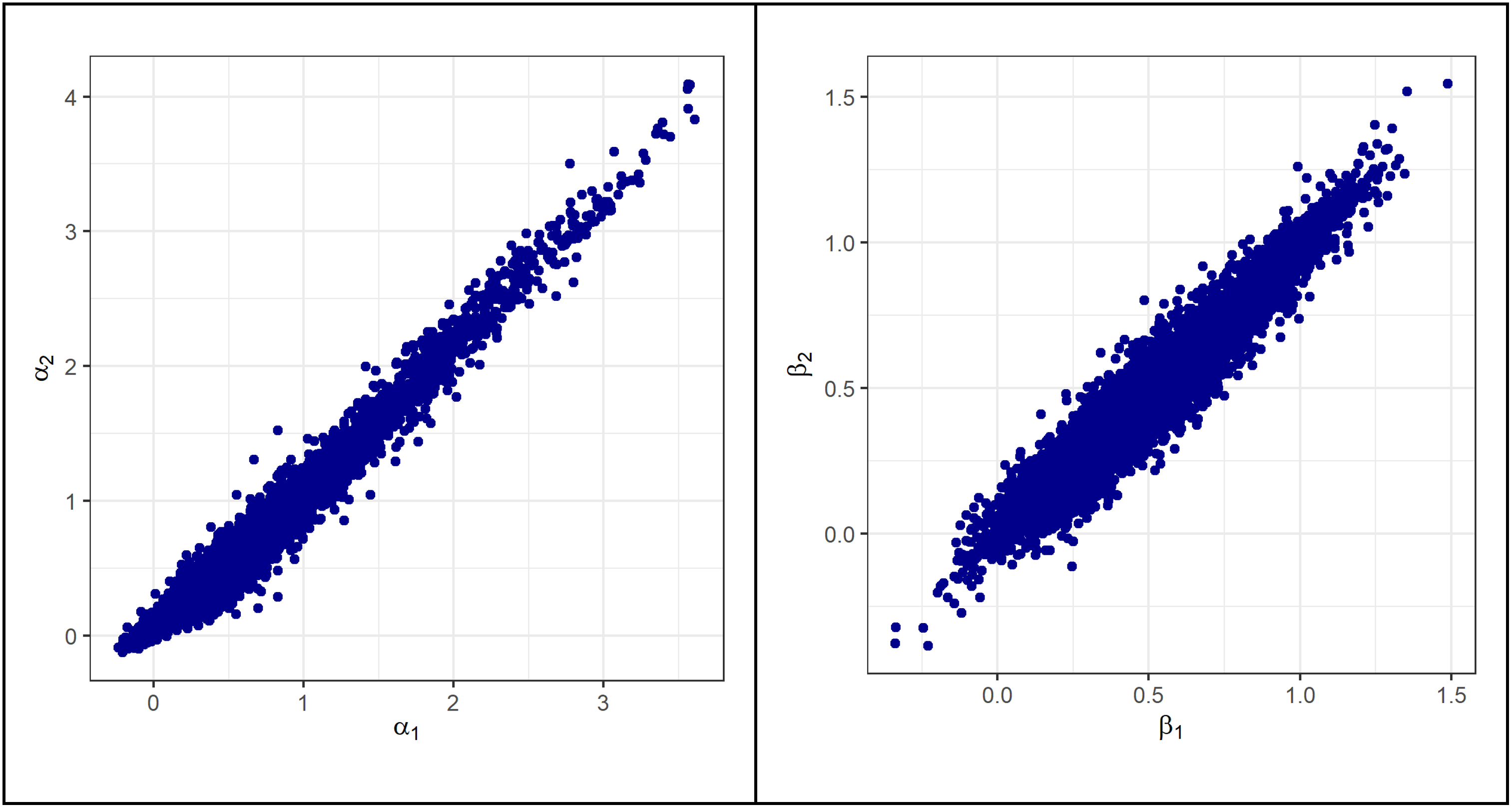

Supplement: Supplementary file 4 — Supporting Information [file PMIC-20-2000009-s003.tif]

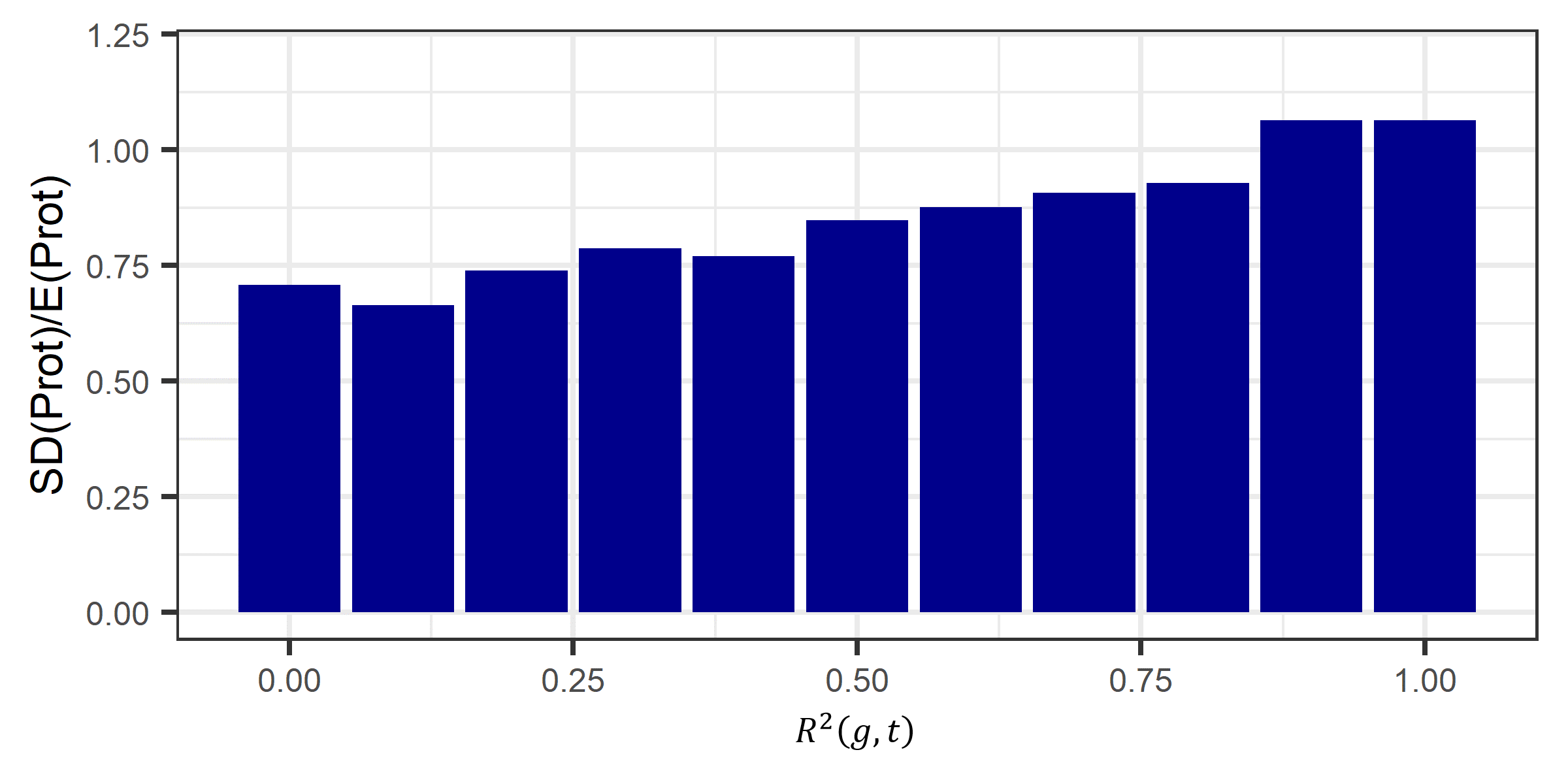

Supplement: Supplementary file 5 — Supporting Information [file PMIC-20-2000009-s004.tif]
