# Supplementary material for: Using Deep Learning to Extrapolate Protein Expression Measurements
Source: Proteomics. 2020 Oct 16;20(21-22):2000009. doi: 10.1002/pmic.202000009 (PMC7757209; doi:10.1002/pmic.202000009)
Supplement: Supplementary file 9 — Supporting Information [file PMIC-20-2000009-s008.docx]

**Using Deep Learning to Extrapolate Protein Expression Measurements**

# **Supporting information**

# 1 Datasets and data pre-processing

Datasets including protein expression values were obtained from the EBI’s Expression Atlas (<https://www.ebi.ac.uk/gxa/home>).^[1]^ RNA data from Expression Atlas are available in two quantified versions: FPKM (Fragments Per Kilobase Million) and TPM (Transcripts Per Kilobase Million), from which FPKM produced slightly better prediction results and therefore were chosen for analysis. The initial raw data for proteomics data processing were taken from the PRIDE database (<https://www.ebi.ac.uk/pride>).^[2]^

*Tissue13* is based on the first high-throughput proteomics datasets that became available: E-PROT-1 (PRIDE dataset identifiers PXD00561, and its re-analysis dataset PXD002967), which contains data about 14 (30) human tissues and 9,641 (17,294) proteins.^[3][4]^ The RNA expression dataset E-MTAB-2836 contains data about RNA expression in 32 human tissues.^[5]^ *Tissue13* contains proteomics expression data for 13 human tissues and 9,637 genes that are shared by both of these datasets.

*NCI60* corresponds to the dataset E-PROT-25 in Expression Atlas (PRIDE dataset identifier PXD005940). It contains data about 58 human cell lines and 8,024 proteins.^[6]^ The available matching RNA expression datasets are E-MTAB-2706 covering 624 cell lines,^[7]^ E-MTAB-2770 with 934 cell lines (from the Cancer Cell Line Encyclopedia)^[8]^ and E-MTAB-3983 with 457 cell lines (from Sanger Genomics of Drug Sensitivity in Cancer Project). The prediction accuracy from these datasets are very similar, with just slightly higher for E-MATB-2770. E-MATB-2770 also shares the highest number of cell lines with the available proteomics dataset *NCI60* and therefore was chosen. *NCI60* contains data about human 46 cell lines and 8,000 genes.

*Tissue29* is based on proteomics data on 29 human tissues E-PROT-29 (PRIDE dataset identifier PXD010154), with 12,893 protein identifications.^[9]^ The matching RNA dataset is E-MTAB-2836 (used in *Tissue13 analysis)*.

*MouseTissue3* corresponds to dataset E-PROT-13 in Expression Atlas.^[10]^ The available matching RNA expression dataset is E-GEOD-43721.^[11]^ However, it covers only 3 mouse tissues. *MouseTissue3* contains data about 3 mouse tissues and 6,591 genes. The mapping between human and mouse homologous genes was based on data from Ensembl using the BioMart interface. It contained 5,388 homologous gene pairs (redundant human genes mapping to the same mouse gene were discarded).

Protein quantification values were taken directly from the Expression Atlas.^[1]^ The analysis protocol of Expression Atlas is described in the respective publication and is as follows. Raw LC-MS data are taken from the PRIDE database and re-analysed using MaxQuant. The different datasets were searched in individual batches against the UniProtKB/Swiss-Prot protein sequence database (May 2019), appended with sequences of common contaminants provided by MaxQuant. Search parameters were chosen to reflect those used in the original publications. In all cases, carbamidomethylation of cysteine was set as fixed modification and oxidation of methionine was set as variable modification. Enzyme specificity was set also according to the enzymes used in the original study, allowing a maximum of two missed cleavages. MS1 tolerance was set to 10 ppm and MS2 tolerance to 20 ppm for Fourier Transform MS data and 0.4 Da for ion trap MS data. PSM (Peptide Spectrum Match), peptide and protein identification FDR (False Discovery Rate) was set at 1% at each level.

Gene annotations with Gene Ontology (GO) identifiers and UniProt Keywords (KW) were extracted from full protein descriptions downloaded from UniProtKB/Swiss-Prot (<https://www.uniprot.org>) (from the "DR GO" and "KW" fields). GO (but not KW) has a hierarchical structure that can be represented using a directed acyclic graph. If a specific GO term was included in protein description, then apart from the term itself, all its ancestors in this hierarchical structure were included in the gene annotation as well. Only annotations that were present for at least 50 genes in human or mouse protein coding genes (as published on UniProtKB/Swiss-Prot, correspondingly 20,367 and 17,027 genes) were chosen for the predictions. This was done to reduce potential prediction artefacts due to overfitting, although the observed impact of the presence or absence of the remaining annotations was negligible.

For RNA expression data available from Expression Atlas the exact numerical values of gene expression were not considered to be directly comparable between different datasets. However, the average numerical values were similar and ranged roughly between 25 and 35 per cell line and tissue, when only non-zero values were considered. Therefore no additional re-scaling was applied. The recommended threshold value of 1.0 was applied, with lower expression values considered to be zero.

For proteomics data, however, the numerical values varied by many orders of magnitude between the four different datasets. Thus, for consistency reasons, for each of the proteomics datasets the expression values were rescaled by multiplying all the values by a dataset-specific constant that was chosen so that the average numerical values for protein expression were equal to the average numerical values of RNA expression. Such rescaling was applied to already filtered subsets of data containing only information about genes and tissues or cell lines that were shared by both the proteomics and RNAseq datasets. The “zero” expression values for RNA data and not available (N/A) values for proteomics data were not used for computing the average values. The same rescaling coefficients were applied to all the tissues or cell lines within a given dataset.

For predictions, the *log-log* transformation^[12]^ was further applied to both RNA and proteomics data, using natural logarithms and adding a small constant to the initial values in order for the transformation to be applicable also to *zero* values of RNA expression, i.e. (apart from N/A values for proteins) each protein or RNA expression value $v$ was replaced by the value $ln(v+1)$. For proteomics data there is no reliable way to distinguish between non-measured and zero expression values and for each tissue or cell line only a subset of genes with available protein expression data was considered for the predictions. For the *NCI60, Tissue29* and *MouseTissue3* datasets protein expression values were available on average for 75% of genes (with a coverage ranging between 60% to 90% for individual tissues and cell lines). In the case of the *Tissue13* dataset, however, the available proteomics data were sparser – with an average coverage of 35%, ranging between 17% and 61% for the individual tissues.

# 2 Deep Learning network for protein abundance prediction

A Deep Learning neural network was developed that performs protein expression value prediction based on RNA expression values and binary gene annotations (such as GO or KW). The network architecture is depicted in Figure S1.


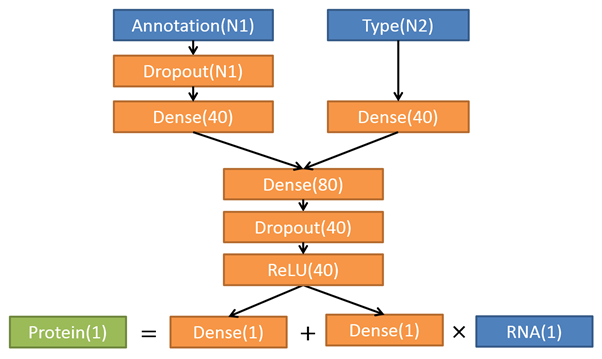


## **Figure S1.** The deep learning network architecture for predicting the protein abundance values based on RNA and gene annotations. Blue boxes represent inputs, the green box outputs, and the orange boxes represent the intermediate layers. In parentheses dimensionalities of input and output vectors as well as those of the network layers are shown.

The neural network calculated the predicted protein expression values of a given gene in a given tissue. The network received three parameters as its inputs – binary gene annotation vector (**Annotation**), tissue or cell type identifier (**Type**) and RNA expression value (**RNA**) with the dimensions correspondingly N1, N2 and 1. It did not receive the explicit gene identifier, so that the network could be used to predict protein abundance for an unknown gene. The gene information, however, was presented indirectly through the annotation vector.

Gene annotation (GO, KW, combined GO and KW, etc.) was encoded as a bit vector where each bit corresponded to the presence or absence of the particular annotation term.

The tissue or cell type identifier was encoded as a one-hot vector having one value of 1 in the position of the column in which the tissue or cell line was provided in the input data matrix and zero values in all other positions.

The neural network was composed of several layers that transformed the inputs to the predicted protein expression values step by step. Gene annotations were compressed into a 40-bit vector by a linear mapping performed by a **Dense** layer. A **Dropout** layer with a dropout rate 0.25 was applied before it to reduce overfitting.^[13]^ This operation helped to reduce overfitting because the vector was several thousands in length, which is of significant length compared to the size of the whole training dataset. Therefore, some form of regularization was beneficial.

Similarly, the tissue or cell type identifier vector was transformed into another 40-bit vector. For combined gene annotation and tissue or cell type data another **Dense** layer was applied to obtain an 80 bit feature vector onto which further passed to **Dropout** and **ReLU** (Rectified Linear Unit) layers, the latter providing a non-linear transformation of data.

As a result, two tissue or cell line specific coefficients called $\alpha(g)$ and $\beta(g)$ were computed and protein abundance values for a given gene *g* with RNA expression value $r(g)$ was calculated according to the formula $p\left( g \right)=\alpha(g)+\beta(g)\times r(g)$. The values $\alpha(g)$ and $\beta\left( g \right)$ therefore were specific for each gene annotation vector and each tissue or cell type. The network allowed for missing tissue type identifiers and/or RNA expression values. Therefore, it could be trained to provide predictions without tissue or cell type information, or predictions based only on gene annotations without using RNA expression data.

The transformation matrices contained in **Dense** layers were dataset-specific and were computed during the network training phase. For this, 90% of the data were used for training and 10% were reserved for testing. Mean squared error (MSE) values between predicted and actual protein abundance values were chosen as the function that was optimized during the training phase. For optimization purposes the Adam optimizer was used with a learning rate of 0.0003.^[14]^ A L2 regularization of weight 0.0001 was also applied to the weight matrices.

The protein abundance level predictions could be obtained directly from the trained network by passing as inputs the gene annotation vector, RNA expression level and the tissue or cell type information (and this was the only option for genes that had not previously been seen by the network during the training phase). Alternatively, the method also allowed the assignment to genes of two gene-specific (or gene- and tissue-specific) coefficients from which prediction of protein expression values could be simply obtained by linear equations. (Assignment of similar gene-specific coefficients had been proposed earlier, but this was done on the basis of extensive wet-lab experiments. In our case these assignments were simply computed *in silico* from provided gene annotations).^[15][16]^ The availability of such gene-specific coefficients also allowed them to test their predictive power for homologous genes, including genes of other organisms. In our experiments we tested the applicability of coefficients computed for human genes for the prediction of protein abundance values in homologous mouse genes.

The network was in Python 3.x programming language and requires TensorFlow library. The current release has been tested with Python version 3.7.8 and TensorFlow version1.15.

# 3 Estimation of prediction accuracy

There are a number of natural estimators that could be used for evaluation of prediction accuracies. Two of these: RMSE (root-mean-square error) and $R^{2}$ (coefficient of determination) can be applied in very general cases, some others, such as Pearson's correlation $r$ or Spearman's correlation $\rho$ are already prediction method specific.

Practically in all related work on protein abundance predictions, however, only $R^{2}$ (sometimes besides $r$ and/or $\rho$) were used. One of apparent reasons for this is that in regression-based models we can simply define $R^{2}=r^{2}$. The other likely reason for not using RMSE is its high dependence on the range of values to which it is applied, making comparisons of results between different datasets difficult. Due to this, the $R^{2}$ score is used consistently here, as is the case for most studies on protein abundance prediction.

Let $X$ be a finite set of (some type) of elements and let $f:X\to R$ and$\hat{f}:X\to R$ be two real valued functions defined for all elements of $X$. (In the context of defining $R^{2}$ score one usually thinks of $f(x)$ as a *measured value* of some parameter associated with element $x\in X$ and of $\hat{f}(x)$ as a *predicted value* of the same parameter.) For an arbitrary total function $\varphi:X\to R$ and an arbitrary subset $Y\subseteq X$ an *unweighted average* of $\varphi(x)$ values over the set $Y$ is denoted by $AVG(\varphi,Y)=\frac{1}{|Y|}\sum_{x\in Y} \varphi(x)$.

The standard definition of prediction accuracy score $R^{2}$ for functions $f$ and$\hat{f}$ on a subset $Y\subseteq X$ is the following:

$R^{2}=1-\frac{\sum_{x\in Y} {(f(x)-\hat{f}(x))}^{2}}{\sum_{x\in Y} {(f(x)-AVG(f,Y))}^{2}}$.

This definition is consistent with $R^{2}$ scores derived from Pearson's correlation, in which case we have $R^{2}=r^{2}$, where $r$ is the Pearson's correlation between the predicted and measured values. In these cases $R^{2}$ ranges from 0 (no prediction) to 1 (perfect prediction). In general case, however, $R^{2}=1$ indicates perfect predictions, but for 'bad predictions' the value can become less than 0. The advantage of an explicit $R^{2}$ definition, however, is the possibility to assign also relative contribution of each gene to the overall $R^{2}$ value, which is further described below.

For each our datasets we are given a set of genes $G$ and a set of tissues $T$ (for brevity here and further we will also refer to cell lines as 'tissues' for datasets that contain cell line data). As a set $X$ in this case we consider a subset of gene and tissue pairs $S\subseteq\left\{ g\in G,t\in T \right\}$ for which measured protein concentrations are available, the functions $f$ and$\hat{f}$ correspond accordingly to the measured values of protein concentrations $p((g,t))$ and the predicted values of protein concentrations $\hat{p}((g,t))$. For DL based predictions the predicted values slightly vary between different network ‘runs’ (with Standard Deviation values ranging between 0.04 and 0.06 for the considered datasets) and we take as $\hat{p}((g,t))$ the average of all the available values (in this study the values predicted by DL models are obtained from averaging the results of 10 different runs of the network for prediction of each particular value).

It will be also useful to consider tissue type specific subsets of $S$: for each $t\in T$ we consider a subset $S[t]=\{(g,u)\in S|u=t\}$. Obviously, all subsets $S[t]$ are disjoint and we have $S=\bigcup_{t\in T} S[t]$. However, due to different data availability for different tissues sets $S[t]$ usually will contain different subsets of genes and will have different sizes.

There are two natural options how to estimate the average score of predictions across all the tissues in a given dataset. Firstly, we can simply apply $R^{2}$ formula for the whole set of gene and tissue pairs $S$ and functions $p$ and$\hat{p}$. We refer to this value as $R_{Davg}^{2}$ – the *average score over the whole dataset*. (The averaging in this case is achieved implicitly by computing $R^{2}$ value for the set $S$ that includes prediction data for all the tissues from the dataset).

Alternatively, we can compute tissue specific prediction accuracy scores $R^{2}[t]$ by applying $R^{2}$ formula for each of the tissue specific sets $S[t]\subseteq S$. The *average score over all tissues* then can be naturally defined as $R_{Tavg}^{2}=\frac{1}{|T|}\sum_{t\in T} R^{2}[t]$. The values of $R_{Tavg}^{2}$ and $R_{Davg}^{2}$ are roughly similar, but not necessarily equal, partially due to the variability in the proportion of N/A values for the different tissues. $R_{Tavg}^{2}$ better corresponds to the intuitive notion of ‘average prediction accuracy’, whilst $R_{Davg}^{2}$ is more directly related to the performance of the DL network since all the genes and tissues are jointly used in one training set.

We also use $R_{Max}^{2}=R^{2}[t]$ and $R_{Min}^{2}=R^{2}[t]$ to refer to the *highest* and *lowest* prediction accuracies for tissues from a given dataset, respectively.

The prediction accuracy for a specific gene and tissue pair $\left( g,t \right)\in S$ is denoted by $R^{2}\left( g,t \right)$ and defined as:

$R^{2}\left( g,t \right)=1-\frac{{(p((g,t))-\hat{p}((g,t)))}^{2}}{\frac{1}{|S|}\sum_{(h,u)\in S} {(p((h,u))-AVG(p,S))}^{2}}$.

Since $R_{Davg}^{2}=\frac{1}{|S|}\sum_{(g,t)\in S} R^{2}\left( g,t \right)$, then $R^{2}\left( g,t \right)$ can be regarded as a natural measure of relative contribution to prediction accuracy for gene $g$ and tissue $t$ to the overall prediction accuracy for all the gene and tissue pairs from $S$. For a particular tissue $t\in T$ the contribution of specific gene $g$ to prediction accuracy $R^{2}[t]$ is denoted by $R^{2}[t]\left( g \right)$ and is computed by similar formula as for $R^{2}\left( g,t \right)$ above, with the value of $t$ being fixed and set $S$ being replaced by $S[t]$. Similarly we have $R^{2}[t]=\frac{1}{|S[t]|}\sum_{(g,t)\in S[t]} R^{2}[t]\left( g \right)$.

The *average* contribution of a gene $g$ to prediction accuracy *across the all tissues* is denoted by $R_{avg}^{2}\left( g \right)$ and defined as:

$R_{avg}^{2}\left( g \right)=\frac{1}{|S(g)|}\sum_{(g,t)\in S(g)} R^{2}\left( g,t \right)$, where $S(g)=\{(h,t)\in S|h=g\}$ is a subset of $S$ that consists of gene and tissue pairs containing the gene $g$.

# 4 Supplementary figures and discussion of the results

Figure S2 shows the percentage of proteins from the three human datasets that could be predicted with $R^{2}\left( g,t \right)$ and $R_{avg}^{2}\left( g \right)$ scores above a certain level within extended (in comparison to Figure 2 in the main text) ranges going from −2.0 to 1.0. This better illustrates the fact that all the curves eventually approach the 100% mark, although the exact $R^{2}$ values for reaching the 100% mark were still lower and range between −30 and −10. A feature to notice for the *NCI60* and *Tissue29* datasets is that within a small range of low $R^{2}$ values, the prediction accuracy by *Randomised* model becomes equal or even better than for the *LR* model. This can be explained by the fact that without providing any information that might be useful for predictions, the DL network assigns for all the genes the predicted values that are close to average protein abundances and thus the 'predictions' turn out to be reasonably good for proteins for which these values are close to average.

| $R^{2}\left( g,t \right)$ | $R_{avg}^{2}\left( g \right)$ |
| --- | --- |
| **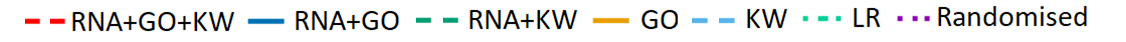** | |
| **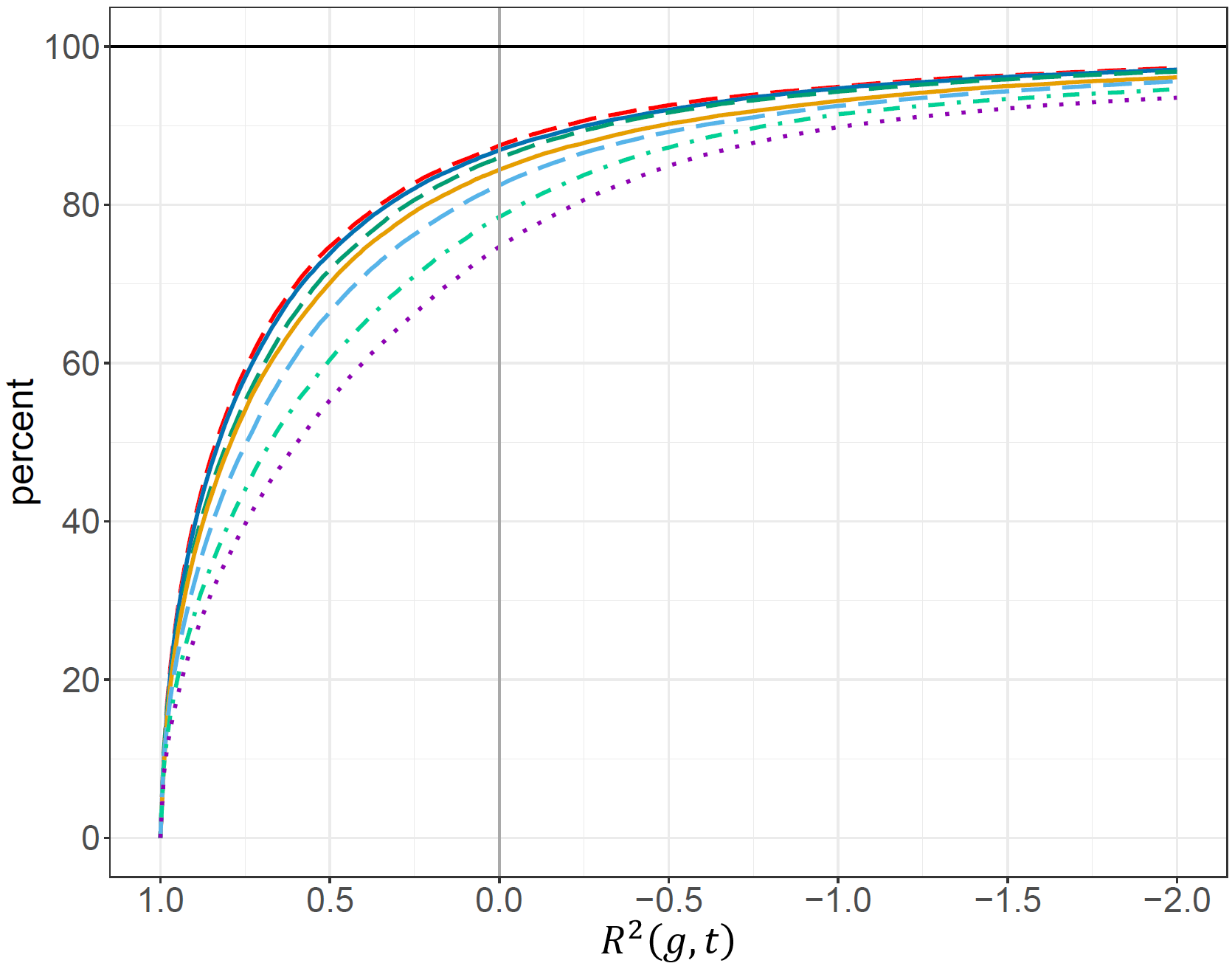** | **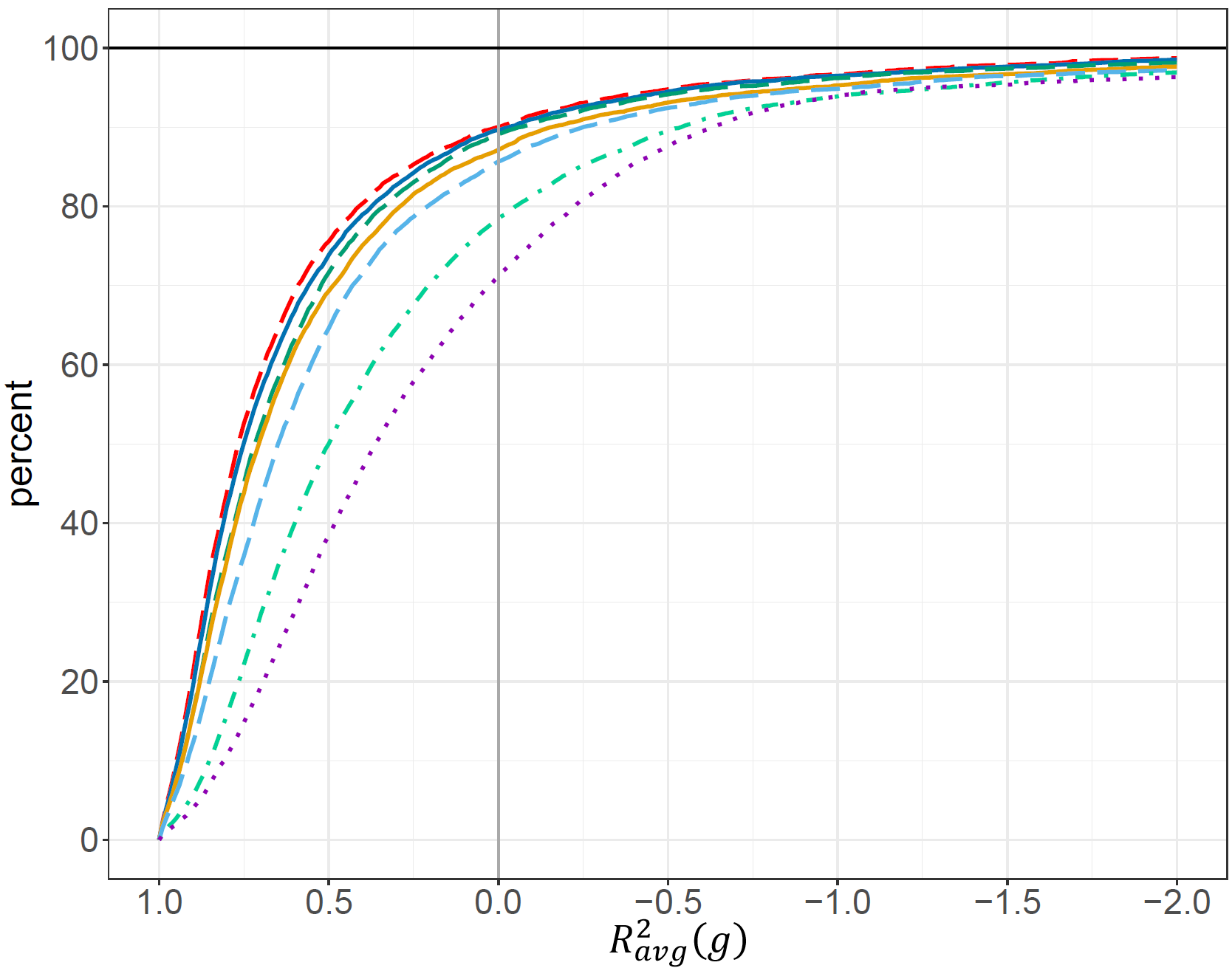** |
| *Tissue13* | |
| **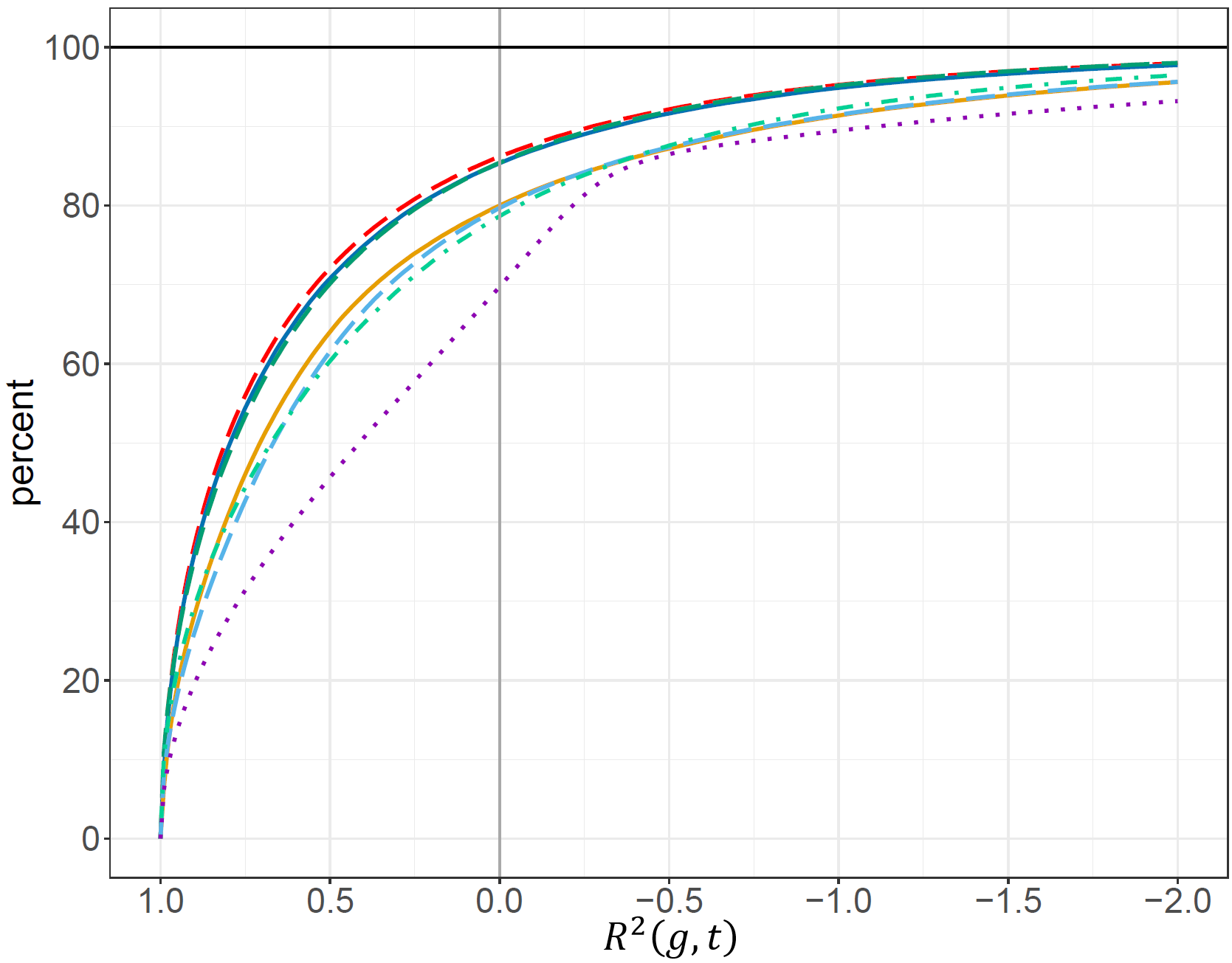** | **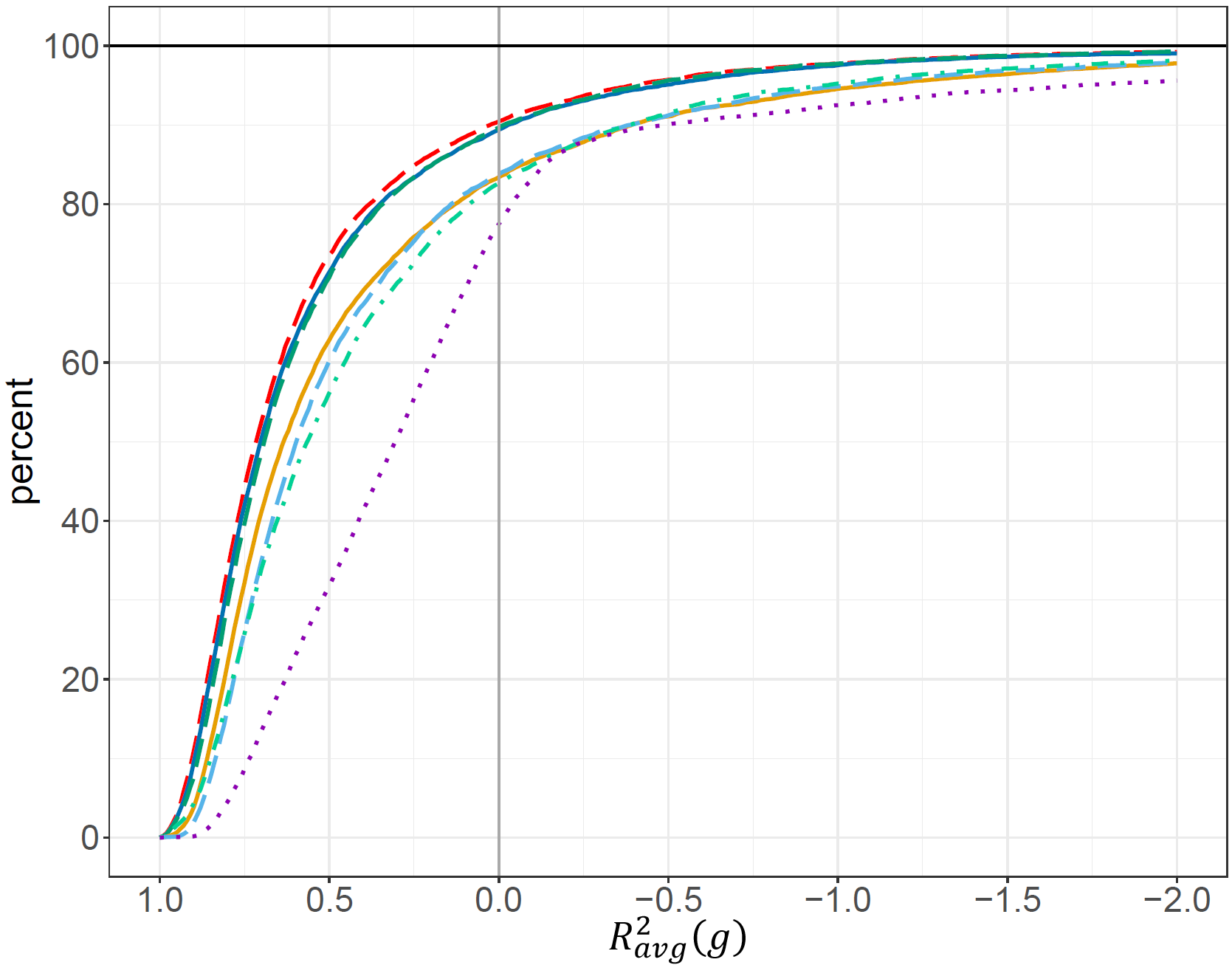** |
| *NCI60* | |
| **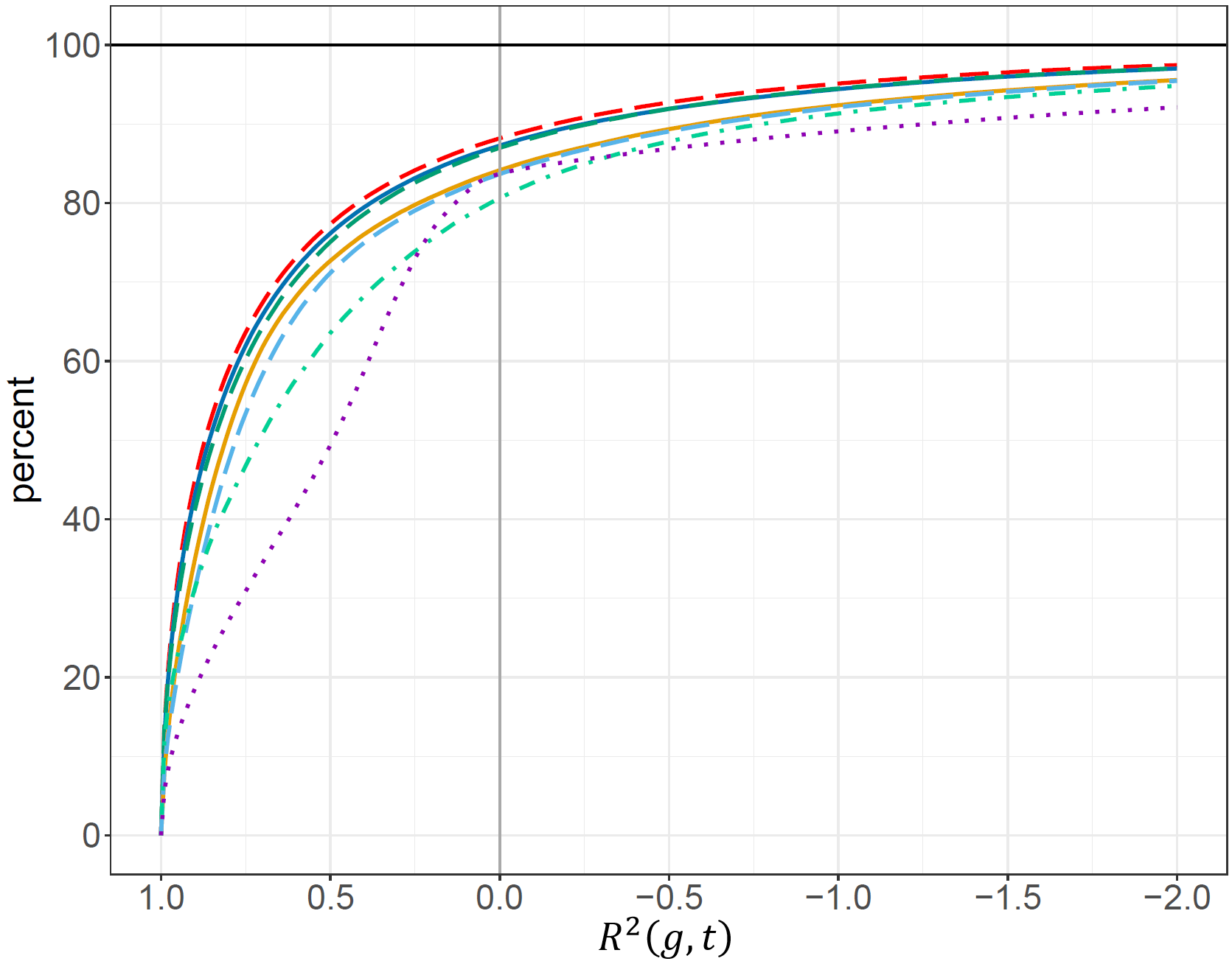** | **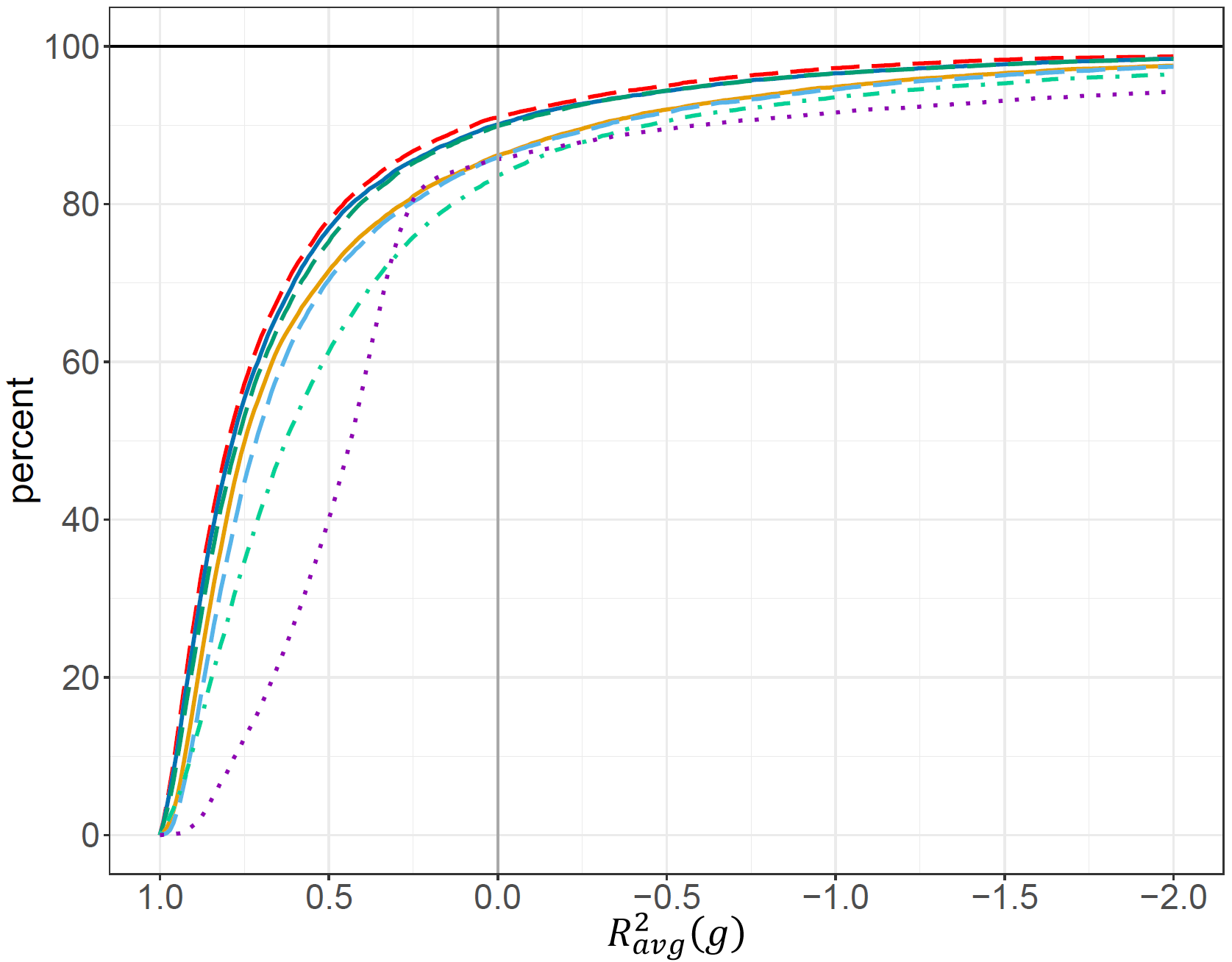** |
| *Tissue29* | |

**Figure S2.** Percentages of genes (on the *y* axes) that could be predicted above $R^{2}$ thresholds (on the *x* axes) within the range $[-2.0,1.0]$. Data are shown for all three human datasets and the scores $R^{2}\left( g,t \right)$ and $R_{avg}^{2}\left( g \right)$. The parts of charts on the left from vertical lines at $R^{2}=0$ cover the data that are shown in Figure 2 in the main text.

Figure S3 shows the prediction accuracies for the *RNA+KW+GO* model for tissues from the *Tissue13* and *Tissue29* datasets, for which correspondingly the most and the least accurate prediction values were obtained (testis and esophagus for *Tissue13* and liver and bone marrow for *Tissue29*). We have not depicted such results for the dataset *NCI60*, since prediction accuracies were more similar for these cell lines, apart from two 'bad’ outliers: cell lines HCT116 and X7860, which are most likely explained by the lower accuracy of proteomics data.

| $R^{2}[t]\left( g \right)$ for *Tissue13* | $R^{2}[t]\left( g \right)$ for *Tissue29* |
| --- | --- |
| 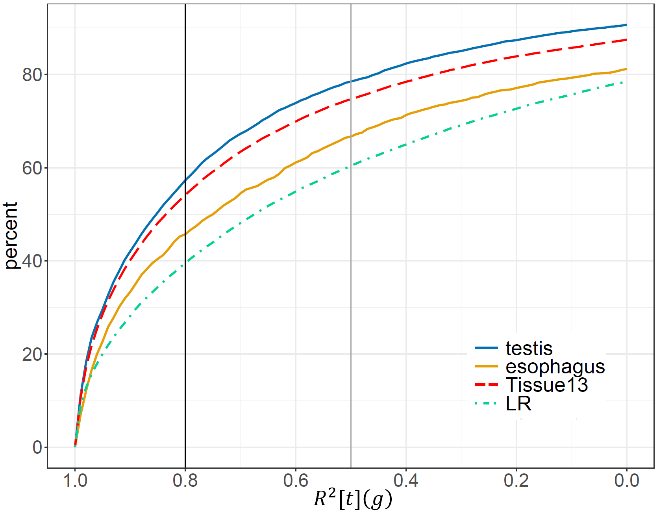 | 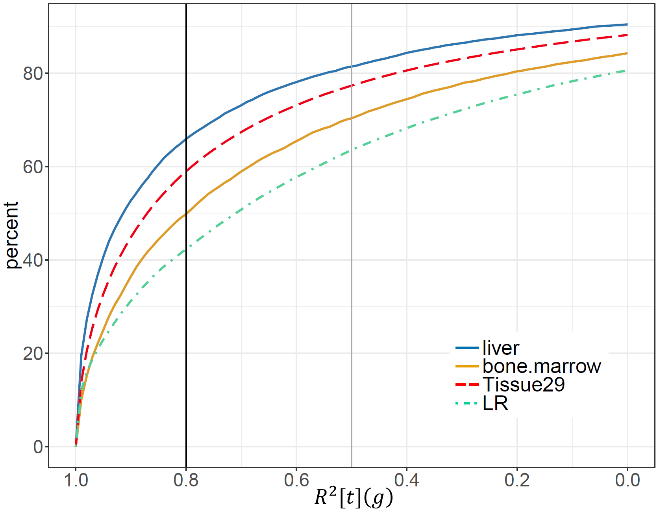 |

**Figure S3.** The performance of the RNA+KW+GO model on the best and worst predictable tissues from the *Tissue13* and *Tissue29* datasets.

Figure S4 shows the variability of the prediction coefficients $\alpha(g)$ and $\beta\left( g \right)$ assigned to genes on the *Tissue29* dataset by two different runs of the RNA+GO+KW model. The assignment of the coefficients was very stable (correlations between assigned $\alpha(g)$ and $\beta\left( g \right)$ values were correspondingly 0.99 and 0.96, respectively). When limited to coefficients assigned to a single sample, such a result mainly might indicate only a practically useful feature of the prediction model. However, since the assigned coefficients can be successfully applied to homologous genes in another sample, the stability of the $\alpha(g)$ and $\beta\left( g \right)$ values already suggest that they might represent the gene-specific contribution of RNA expression values to protein abundance values.

| 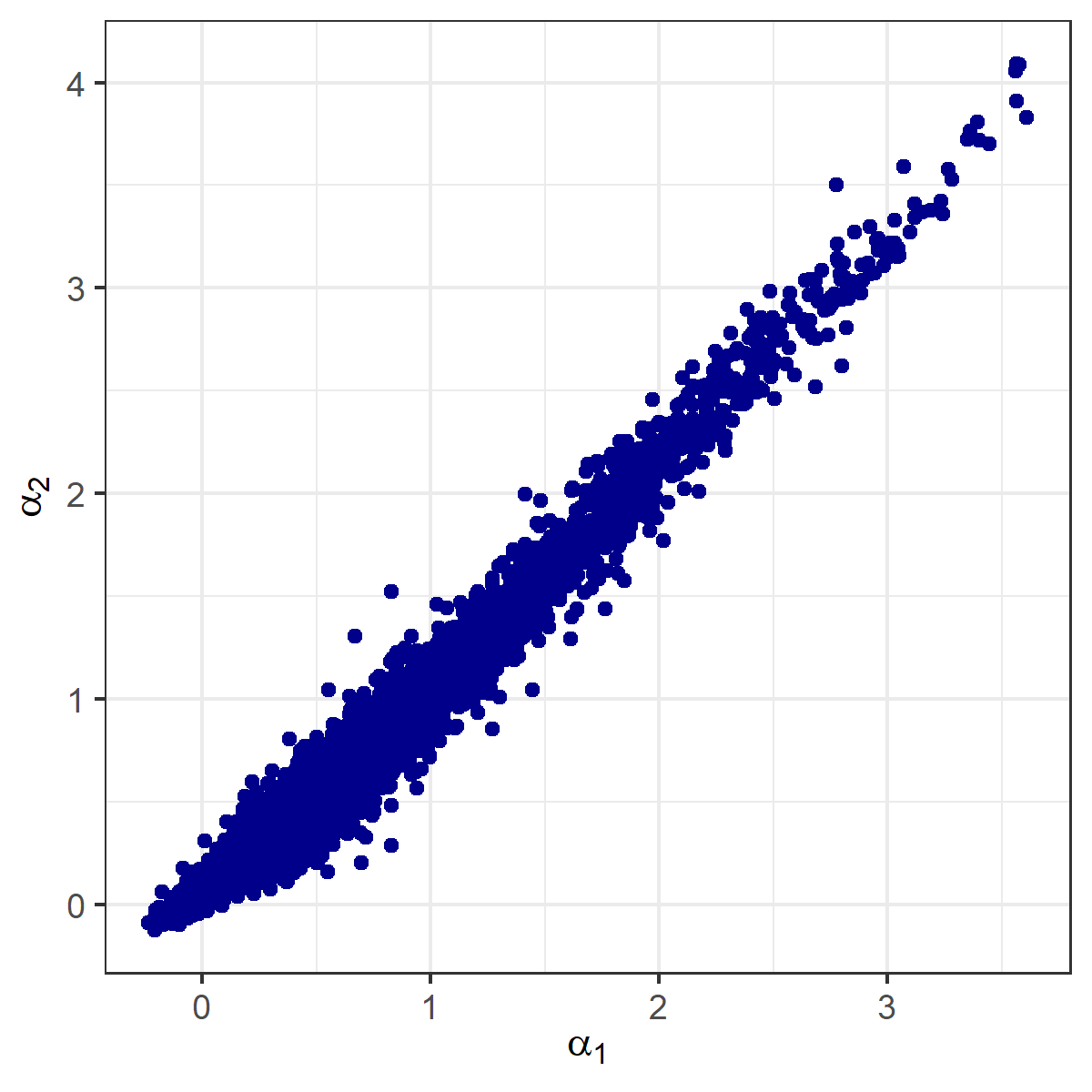 | 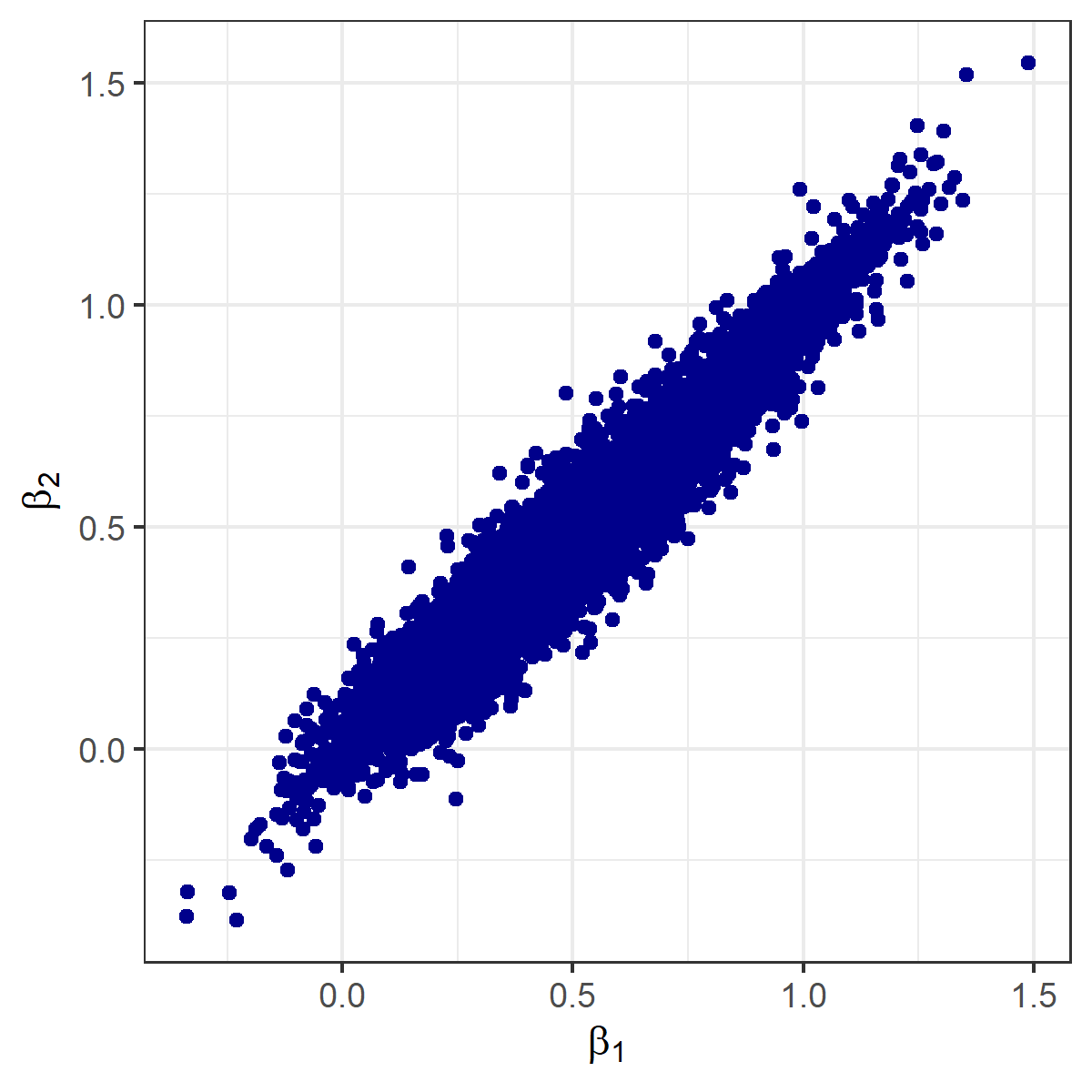 |
| --- | --- |

**Figure S4.** Variability of the prediction coefficients $\alpha(g)$ and $\beta\left( g \right)$ assigned to genes on the *Tissue29* dataset by two different runs of the RNA+GO+KW model. The corresponding correlations between the $\alpha(g)$ and $\beta\left( g \right)$ values were 0.99 and 0.96*,* respectively.

Figure S5 shows the relationship between the prediction accuracy $R^{2}\left( g,t \right)$ and the relative variability of protein concentrations among the tissues (SD divided by mean protein abundance value) for the *Tissue29* dataset. This is a different representation of the observation shown in Figure 3 (in the main text) summarising the fact that better predictions were obtained for proteins with a larger abundance variability between different tissues. A possible explanation is that the concentrations of such proteins were more affected by the levels of RNA expression. As it can be seen, such a trend, whilst noticeable, was not particularly strong.


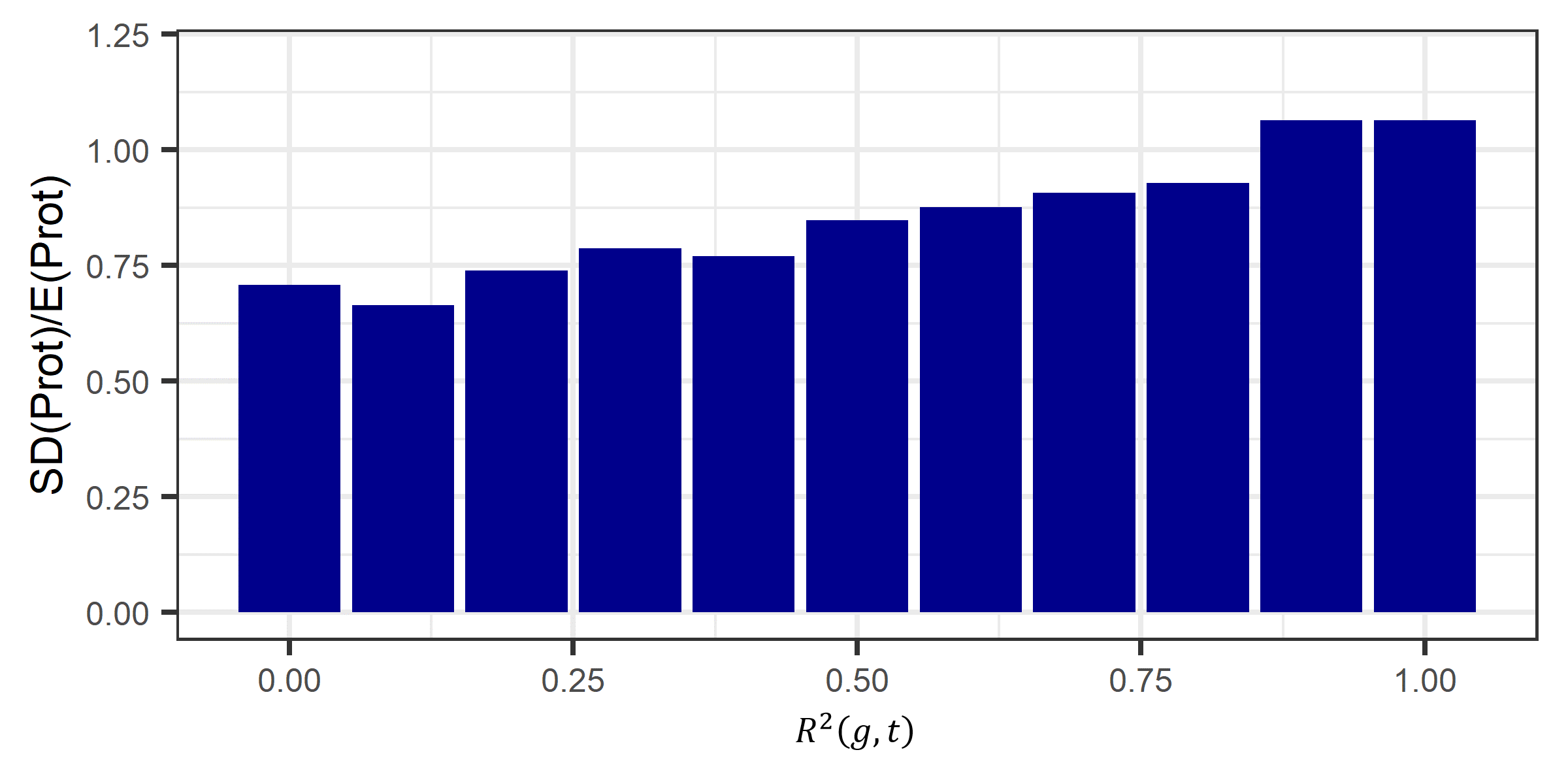
**Figure S5.** Relation between the prediction accuracy $R^{2}\left( g,t \right)$ and the relative variability of protein concentrations among tissues (SD divided by mean protein abundance values) for the *Tissue29* dataset. The proteins with prediction accuracies between 0.0 and 1.0 were grouped into 10 accuracy intervals of equal length. The vertical bar for each interval represents the average relative variability of protein concentrations from that interval.

# 5 Supplementary tables

Three supplementary tables (in Microsoft Excel format) are provided as Supplementary Material and are available from GitHub repository.

*Supp_Table_1_Predictions* contains prediction accuracy data – the obtained $R^{2}$ values for each of the four datasets, all tissues and cell types in these datasets, and all prediction models (i.e. information provided to DL network) tested on each specific dataset.

*Supp_Table_2_HumanToMouseCoefficients* contains the mouse gene (but not tissue) specific coefficients assigned to 9,699 homologous human genes from the *Tissue29* dataset for the two tissues (liver and testis) that are shared with the *MouseTissue13* dataset. From these 9,699 genes, 5,388 of them were present in the *MouseTissue13* dataset. This is the most predictive set of coefficients obtained from the network trained on RNA expression and combined GO and KW annotation data (tissue specificity was not included in the training data since it did not provide improved predictions).

*Supp_Table_3_AnnotationTermAccuracies* contains the information about contribution to prediction accuracy of specific GO and KW terms.

# 6 Software and dataset availability

The developed software is available in a GitHub repository: <https://github.com/IMCS-Bioinformatics/DLNetworkForProteinAbundancePrediction>. The repository contains programs implementing a DL network for protein abundance prediction and also a program for preparing subsets of data (genes and tissues or cell lines) that are shared by two given protein abundance and RNA expression datasets and for rescaling protein abundance values to be consistent with RNA expression values. The software is implemented in Python 3.x programming language and requires TensorFlow library. The current release has been tested with Python version 3.7.8 and TensorFlow version1.15.

As mentioned above, the original quantitative proteomics and RNA expression datasets used for this study are available from Expression Atlas (<https://www.ebi.ac.uk/gxa/home>). The GitHub repository contains the pre-processed *Tissue13*, *NCI60*, *Tissue29* and *MouseTissue3* datasets as well as the gene annotation files suitable for input to the abundance prediction network. The repository also contains the *Supp_Table_1_Predictions*, *Supp_Table_2_HumanToMouseCoefficients* and *Supp_Table_3_AnnotationTermAccuracies* files, including a brief summary of the results and the prediction coefficients that were assigned to mouse genes on the basis of their homology to human genes.

# References

[1] I. Papatheodorou, P. Moreno, J. Manning, A. M. P. Fuentes, N. George, S. Fexova, N. A. Fonseca, A. Füllgrabe, M. Green, N. Huang, L. Huerta, H. Iqbal, M. Jianu, S. Mohammed, L. Zhao, A. F. Jarnuczak, S. Jupp, J. Marioni, K. Meyer, R. Petryszak, C. A. Prada Medina, C. Talavera-López, S. Teichmann, J. A. Vizcaino, A. Brazma, Nucleic Acids Res. 2020, 48, D77.

[2] Y. Perez-Riverol, A. Csordas, J. W. Bai, M. Bernal-Llinares, S. Hewapathirana, D. J. Kundu, A. Inuganti, J. Griss, G. Mayer, M. Eisenacher, E. Perez, J. Uszkoreit, J. Pfeuffer, T. Sachsenberg, S. Yilmaz, S. Tiwary, J. Cox, E. Audain, M. Walzer, A. F. Jarnuczak, T. Ternent, A. Brazma, J. A. Vizcaino, Nucleic Acids Res. 2019, 47, D442.

[3] M. S. Kim, S. M. Pinto, D. Getnet, R. S. Nirujogi, S. S. Manda, R. Chaerkady, A. K. Madugundu, D. S. Kelkar, R. Isserlin, S. Jain, J. K. Thomas, B. Muthusamy, P. Leal-Rojas, P. Kumar, N. A. Sahasrabuddhe, L. Balakrishnan, J. Advani, B. George, S. Renuse, L. D. N. Selvan, A. H. Patil, V. Nanjappa, A. Radhakrishnan, S. Prasad, T. Subbannayya, R. Raju, M. Kumar, S. K. Sreenivasamurthy, A. Marimuthu, G. J. Sathe, S. Chavan, K. K. Datta, Y. Subbannayya, A. Sahu, S. D. Yelamanchi, S. Jayaram, P. Rajagopalan, J. Sharma, K. R. Murthy, N. Syed, R. Goel, A. A. Khan, S. Ahmad, G. Dey, K. Mudgal, A. Chatterjee, T. C. Huang, J. Zhong, X. Y. Wu, P. G. Shaw, D. Freed, M. S. Zahari, K. K. Mukherjee, S. Shankar, A. Mahadevan, H. Lam, C. J. Mitchell, S. K. Shankar, P. Satishchandra, J. T. Schroeder, R. Sirdeshmukh, A. Maitra, S. D. Leach, C. G. Drake, M. K. Halushka, T. S. K. Prasad, R. H. Hruban, C. L. Kerr, G. D. Bader, C. A. Iacobuzio-Donahue, H. Gowda, A. Pandey, Nature 2014, 509, 575.

[4] J. C. Wright, J. Mudge, H. Weisser, M. P. Barzine, J. M. Gonzalez, A. Brazma, J. S. Choudhary, J. Harrow, Nat. Commun. 2016, 7.

[5] M. Uhlen, L. Fagerberg, B. M. Hallstrom, C. Lindskog, P. Oksvold, A. Mardinoglu, A. Sivertsson, C. Kampf, E. Sjostedt, A. Asplund, I. Olsson, K. Edlund, E. Lundberg, S. Navani, C. A. Szigyarto, J. Odeberg, D. Djureinovic, J. O. Takanen, S. Hober, T. Alm, P. H. Edqvist, H. Berling, H. Tegel, J. Mulder, J. Rockberg, P. Nilsson, J. M. Schwenk, M. Hamsten, K. von Feilitzen, M. Forsberg, L. Persson, F. Johansson, M. Zwahlen, G. von Heijne, J. Nielsen, F. Ponten, Science 2015, 347.

[6] A. M. Gholami, H. Hahne, Z. X. Wu, F. J. Auer, C. Meng, M. Wilhelm, B. Kuster, Cell Reports 2013, 4, 609.

[7] C. Klijn, S. Durinck, E. W. Stawiski, P. M. Haverty, Z. S. Jiang, H. B. Liu, J. Degenhardt, O. Mayba, F. Gnad, J. F. Liu, G. Pau, J. Reeder, Y. Cao, K. Mukhyala, S. K. Selvaraj, M. M. Yu, G. J. Zynda, M. J. Brauer, T. D. Wu, R. C. Gentleman, G. Manning, R. L. Yauch, R. Bourgon, D. Stokoe, Z. Modrusan, R. M. Neve, F. J. de Sauvage, J. Settleman, S. Seshagiri, Z. M. Zhang, Nat. Biotechnol. 2015, 33, 306.

[8] J. Barretina, G. Caponigro, N. Stransky, K. Venkatesan, A. A. Margolin, S. Kim, C. J. Wilson, J. Lehar, G. V. Kryukov, D. Sonkin, A. Reddy, M. W. Liu, L. Murray, M. F. Berger, J. E. Monahan, P. Morais, J. Meltzer, A. Korejwa, J. Jane-Valbuena, F. A. Mapa, J. Thibault, E. Bric-Furlong, P. Raman, A. Shipway, I. H. Engels, J. Cheng, G. Y. K. Yu, J. J. Yu, P. Aspesi, M. de Silva, K. Jagtap, M. D. Jones, L. Wang, C. Hatton, E. Palescandolo, S. Gupta, S. Mahan, C. Sougnez, R. C. Onofrio, T. Liefeld, L. MacConaill, W. Winckler, M. Reich, N. X. Li, J. P. Mesirov, S. B. Gabriel, G. Getz, K. Ardlie, V. Chan, V. E. Myer, B. L. Weber, J. Porter, M. Warmuth, P. Finan, J. L. Harris, M. Meyerson, T. R. Golub, M. P. Morrissey, W. R. Sellers, R. Schlegel, L. A. Garraway, Nature 2012, 483, 603.

[9] D. X. Wang, B. Eraslan, T. Wieland, B. Hallstrom, T. Hopf, D. P. Zolg, J. Zecha, A. Asplund, L. H. Li, C. Meng, M. Frejno, T. Schmidt, K. Schnatbaum, M. Wilhelm, F. Ponten, M. Uhlen, J. Gagneur, H. Hahne, B. Kuster, Molecular Systems Biology 2019, 15.

[10] E. L. Huttlin, M. P. Jedrychowski, J. E. Elias, T. Goswami, R. Rad, S. A. Beausoleil, J. Villen, W. Haas, M. E. Sowa, S. P. Gygi, Cell 2010, 143, 1174.

[11] M. Soumillon, A. Necsulea, M. Weier, D. Brawand, X. L. Zhang, H. C. Gu, P. Barthes, M. Kokkinaki, S. Nef, A. Gnirke, M. Dym, B. de Massy, T. S. Mikkelsen, H. Kaessmann, Cell Reports 2013, 3, 2179.

[12] A. L. Koch, J. Theor. Biol. 1966, 12, 276.

[13] N. Srivastava, G. Hinton, A. Krizhevsky, I. Sutskever, R. Salakhutdinov, J. Mach. Learn. Res. 2014, 15, 1929.

[14] D. P. Kingma, J. L. Ba, in *ICLR 2015*, International Conference on Learning Representations, ICLR, 2015.

[15] F. Edfors, F. Danielsson, B. M. Hallstrom, L. Kall, E. Lundberg, F. Ponten, B. Forsstrom, M. Uhlen, Molecular Systems Biology 2016, 12.

[16] B. Eraslan, D. X. Wang, M. Gusic, H. Prokisch, B. M. Hallstrom, M. Uhlen, A. Asplund, F. Ponten, T. Wieland, T. Hopf, H. Hahne, B. Kuster, J. Gagneur, Molecular Systems Biology 2019, 15.
